# Patient selection for CAR T or BiTE therapy in multiple myeloma: Which treatment for each patient?

**DOI:** 10.1186/s13045-022-01296-2

**Published:** 2022-06-07

**Authors:** David Kegyes, Catalin Constantinescu, Louise Vrancken, Leo Rasche, Celine Gregoire, Bogdan Tigu, Diana Gulei, Delia Dima, Alina Tanase, Hermann Einsele, Stefan Ciurea, Ciprian Tomuleasa, Jo Caers

**Affiliations:** 1 Medfuture Research Center for Advanced Medicine, Iuliu Hatieganu University of Medicine and Pharmacy, Cluj-Napoca, Romania; 2 Department of Hematology, Iuliu Hatieganu University of Medicine and Pharmacy, Cluj-Napoca, Romania; 3 Department of Hematology, Ion Chiricuta Clinical Cancer Center, Cluj-Napoca, Romania; 4 Laboratory of Hematology, University of Liège, Liège, Belgium; 5 Department of Hematology, CHU de Liège, Liège, Belgium; 6 Department of Stem Cell Transplantation, Fundeni Clinical Institute, Bucharest, Romania; 7 Department of Internal Medicine II, University of Würzburg, Würzburg, Germany; 8 Hematopoietic Stem Cell Transplantation and Cellular Therapy Program, Division of Hematology/Oncology, Chao Family Comprehensive Cancer Center, University of California, Irvine, USA

**Keywords:** Multiple myeloma, Immunotherapy, Adoptive cell therapy, Chimeric antigen receptor, CAR T, Bispecific T cell engager, BiTE, Bispecific antibody, Stem cell transplantation, Bispecific antibody armed T cell, BAT

## Abstract

Multiple myeloma (MM) is a plasma cell malignancy that affects an increasing number of patients worldwide. Despite all the efforts to understand its pathogenesis and develop new treatment modalities, MM remains an incurable disease. Novel immunotherapies, such as CAR T cell therapy (CAR) and bispecific T cell engagers (BiTE), are intensively targeting different surface antigens, such as BMCA, SLAMF7 (CS1), GPRC5D, FCRH5 or CD38. However, stem cell transplantation is still indispensable in transplant-eligible patients. Studies suggest that the early use of immunotherapy may improve outcomes significantly. In this review, we summarize the currently available clinical literature on CAR and BiTE in MM. Furthermore, we will compare these two T cell-based immunotherapies and discuss potential therapeutic approaches to promote development of new clinical trials, using T cell-based immunotherapies, even as bridging therapies to a transplant.

## Background on current therapies in multiple myeloma (MM)

**Fig. 1:**
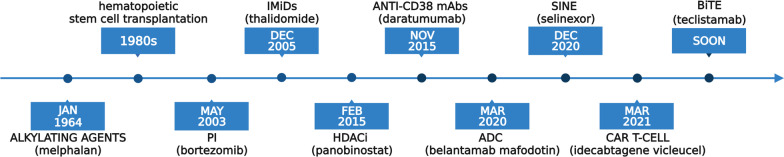
FDA-approved therapeutical options for MM and date of their first approval. PI—proteasome inhibitors, IMiDs—immunomodulatory drugs, HDACi—histone deacetylase inhibitors, mAbs—monoclonal antibodies, ADC—antibody–drug conjugates, SINEs—selective inhibitors of nuclear export, CAR—CAR T cell therapies, BiTE—bispecific T cell engagers

MM is a common hematologic malignancy and an aggressive plasma cell dyscrasia, causing the death of approximately 106,000 people worldwide each year. [1] Due to the aging population, its global burden is constantly rising, with the highest incidence reported in Australia, Western Europe, and the USA. As a multifactorial disorder, MM is characterized by clinical and molecular heterogeneity. Its symptomatic phase is preceded by a premalignant monoclonal gammopathy of undetermined significance (MGUS) followed by a malignant but asymptomatic phase, called smoldering multiple myeloma. These precursor stages are defined by clear diagnostic criteria. [1] Genetic alterations, mostly translocations and hyperdiploidy, play the most significant role in its pathogenesis [2]. Genetic aberrations dysregulate the cancer-immunity cycle, resulting in hampered immune surveillance and uncontrolled cell proliferation [3]. Regarding therapeutic options, as shown in Fig. 1, the last two decades have refashioned MM treatment dramatically. Novel standard of care regimens were implemented that include proteasome inhibitors (PI) (e.g., bortezomib, carfilzomib, ixazomib), immunomodulatory drugs (IMiDs) (e.g., thalidomide, lenalidomide, pomalidomide), histone deacetylase inhibitors (HDACi) (e.g., panobinostat), monoclonal antibodies (mABs) (e.g., anti-CD38 mAbs daratumumab and isatuximab, anti-CS1 mAb elotuzumab), antibody–drug conjugates (ADC) (e.g., belantamab mafodotin) and selective inhibitors of nuclear export (SINEs) (e.g., selinexor) [4, 5].

Even in the era of new agents, autologous stem cell transplantation (SCT) after high-dose melphalan remains a key element in treating newly diagnosed MM [6]. SCT is preceded by induction therapy, in which combinations of PI and IMIDs are typically used. Despite the large number of drug combinations, drug resistance is a well-known phenomenon in MM [7]. The immunosuppressive tumor microenvironment plays a crucial role not only in disease biology but also in drug resistance mechanisms [8]. To overcome both pathophysiological and pharmaceutical challenges, T cell-based immunotherapies, such as CAR T (CAR) or bispecific T cell engager (BiTE), were developed. CAR and BiTE are mostly studied in relapsed/refractory (R/R) disease. Nevertheless, it is suggested that early use of CAR increases treatment efficacy and improves outcomes [9]. In this context, we review and evaluate present data on CAR and BiTE clinical studies in MM to encourage the initiation of future clinical trials, employing T cell-based immunotherapies as a bridging therapy to transplantation.

### CAR

The first CAR structure was engineered in 1989 [10]. Chimeric antigen receptors (CARs) are recombinant membrane proteins, generally transduced ex vivo in T cells, using retroviral vectors [11]. Other ways of generating CAR T cells include the use of non-viral methods such as CRISPR/Cas9 [12] or transposon systems [13, 14], or more recently, in vitro-transcribed mRNA was used to reprogram T cells in vivo [15]. With the help of mRNA, T cells can be created differently than traditional CARs, which use viral vectors to transduce them. Through mRNA transfection, mRNA CARs are engineered to express a protein that can target a specific antigen-binding domain for a defined period of time. It has the advantage of the prevention of uncontrolled cell proliferation. In fact, CD8 + anti-BCMA mRNA CARs are being tested in the DESCARTES 08 trial for RRMM (NCT034488978) [16]. A study by Foster et al. reported that mRNA CARs are a safer alternative to classical CARs that retain the benefits of CARs without the severe side effects; however, there is still a need to find ways to enhance their potency [17].

**Fig. 2:**
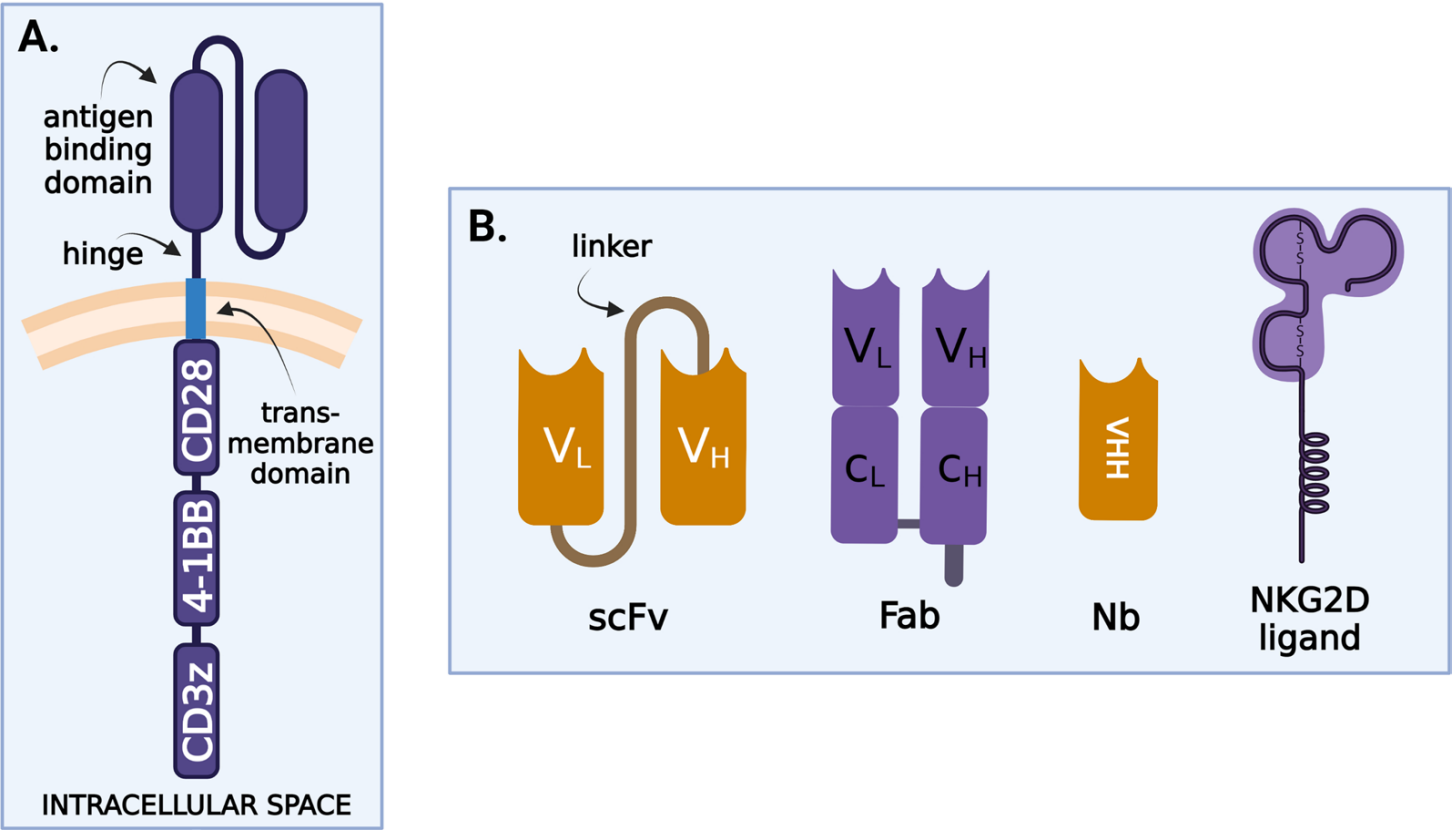
**A** CARs structure. CD3zeta—stimulatory domain, 4-1BB—costimulatory domains; **B** receptor binding domains—scFv—single-chain variable fragments, *F*_ab_—antigen-binding fragment, Nb—nanobody

CARs have two main functions: antigen-binding and MHC-independent T-lymphocyte activation. Structurally (Fig. 2A), CARs are characterized by an extracellular and intracellular region connected via a transmembrane domain. The extracellular region consists of an antigen recognition domain and a hinge region (spacer).

The *antigen recognition domain* confers target antigen specificity. There are four main antigen-binding counterparts (Fig. 2B) with single-chain variable fragments (scFv) the most common. They consist of a variable heavy (*V*_*H*_) and a variable light (*V*_*L*_) chain derived from antibodies and connected via a linker. Second antigen-binding counterparts are antigen-binding fragments (*F*_ab_), antigen-binding regions of natural immunoglobulin structures that consist of *V*_*H*_, *V*_*L*_, and two interconnected constant regions (*C*_*H*_, *C*_*L*_). In the third place, nanobodies, the smallest antibody fragments, retain a full antigen-binding capacity. They are formed by a variable heavy-chain domain (VHH) and are derived from heavy-chain-only antibodies coming from *Camelidae* or sharks. They are the smallest functional antibody fragments [18]. Last, but not least are natural receptors or ligands, found normally on cell surfaces [19]. Such a potent natural receptor in MM is NKG2D. Its role in treating MM will be discussed later.

The *hinge (spacer)* region connects the antigen-binding and transmembrane domains. It provides flexibility to the CARs and determines the intercellular distance, crucial for epitope recognition and the creation of a functional immunological synapse. It is suggested that the length and structure of the hinge affects expression, membrane transport, and the signaling thresholds of CARs [20]. Thus, by modulating signaling threshold, the hinge region is important to modulate on-target off-tumor toxicities too. A spacer is not always required: Its necessity depends on the distance of target epitopes from the cell membrane [21–24]. The majority of CARs targeted against MM cells contain a hinge region derived from short amino acid sequences of CD8 or CD28.

The structure of the *transmembrane domain* is also clinically relevant, not only because it anchors CARs to the T lymphocytes but also because it participates in the transduction of ligand recognition signals to the intracellular domain. Its structure influences CARs’ membrane expression levels and stability, therefore modulating signaling responses [20]. Transmembrane domains in CARs are mostly derived from CD28, CD3zeta, CD8, and CD4 [25].

The *intracellular signaling domain* can be divided into principal stimulatory and secondary costimulatory domains. CD3zeta intracellular stimulatory domain, containing immunoreceptor tyrosine activation motifs (ITAMs) that provide “signal 1” is routinely used in CARs targeting MM antigens. CAR T cells are divided into several categories based on the number of costimulatory domains. First-generation CARs, without costimulatory domains, were not sufficiently effective to be used in clinical practice. The natural ligands, such as the NKG2D CARs, are an exception. They usually have an endogenous, natural costimulatory domain that does not require transduction of another costimulatory domain. The majority of CARs used in MM are second-generation constructs, which contain either a CD28 or a 4-1BB (CD137) costimulatory domain. Although CD28-harboring cells are more potent and have higher expansion capacities, 4-1BB CARs present a memory stem cell-like phenotype, resulting in longer persistence [26]. According to these findings, CARs predominantly carry 4-1BB. Other costimulatory domains, such as OX40 (CD134), CD27, inducible T cell costimulator (ICOS), CD40 or MYD88, were also engineered. In their review, Weinkove et al. [27] outlined key information about the aforementioned costimulation domains, but these have only been studied in preclinical stages. Third-generation CARs contain two or more costimulatory domains, which are intended to improve the effectiveness and persistence of CARs. There is a big difference between dual targeting: a T cell that targets a myeloma cell by having to antigen-binding sites that can both activate the T cells. The true costimulatory CAR T approaches implicate the binding of both antigens for T cell activation. In this case, there is a mild activation that is further enhanced by the costimulatory activation. The disadvantages are that they have a more complex design and development, and it is too early in their development to show a better efficacy compared to single-ag targeting. Also, if tumor cells loose one of the antigens, the costimulatory CAR is no longer activated [28]. Fourth- and fifth-generation MM antigen-targeted CARs have also been generated, which release immunomodulatory molecules (IL-7, CCL19) following antigen stimulation [29].

The interplay of these components leads to the formation of an immunological synapse between tumoral cells and CARs. This process results in killing target cells by multiple pathways and includes the release of cytotoxic molecules (perforins, granzymes), the induction of apoptosis by Fas–Fas ligand molecular pathway and cytokine production, leading to both lymphocyte proliferation and the activation of other immune cells [30].

### BiTE

**Fig. 3:**
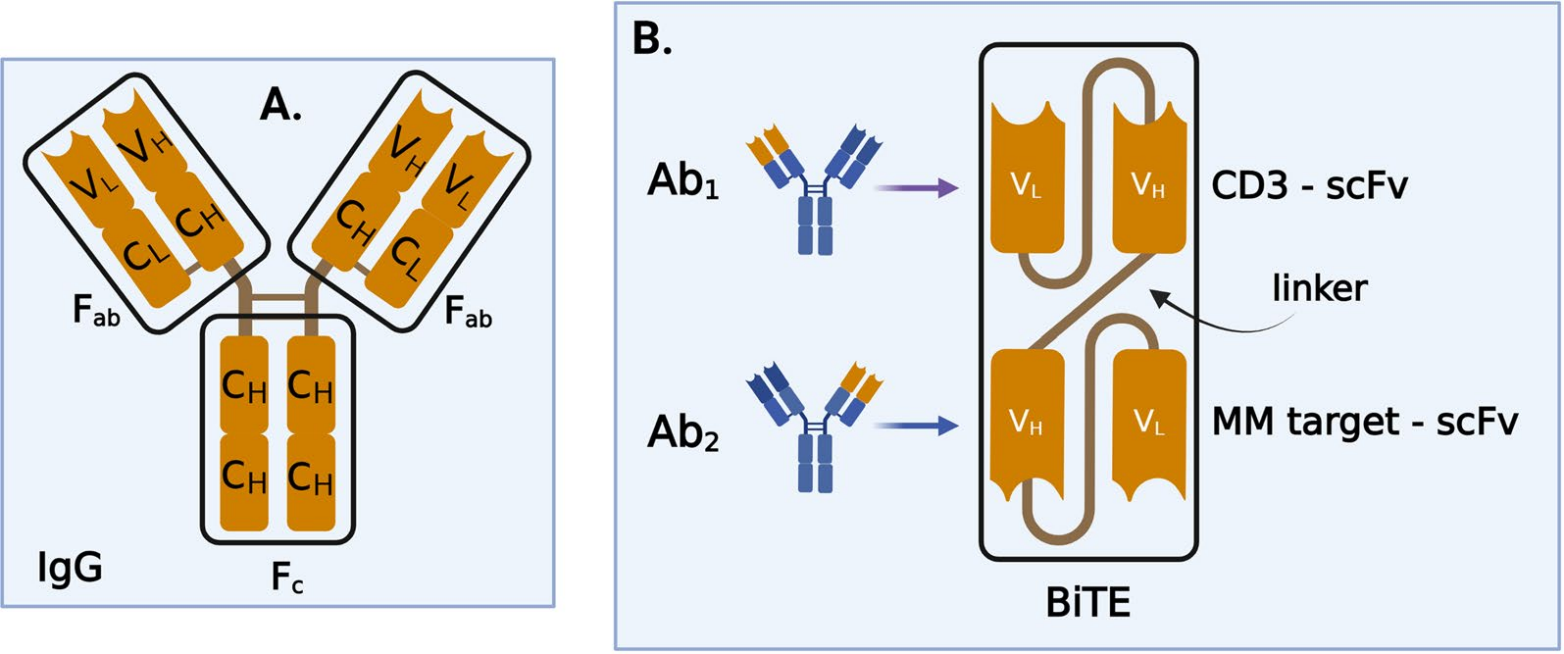
Bispecific antibody types. **A** IgG-like, formed by a crystallizable effector region and two variables, antigen-binding regions. IgG—immunoglobulin G, *F*_*c*_—crystallizable region, *F*_ab_—antigen-binding region, *V*_*L*_—variable light chain, *V*_*H*_—variable heavy chain, *C*_*H*_—constant heavy chain; **B** BiTEs structure. scFv—single-chain variable fragments, Ab1—antibody 1, Ab2—antibody 2

Bispecific antibodies (BsAbs) are small, dual-targeting antibodies. Two main structural, antigen-binding forms of BsAbs are distinguished: immunoglobulin G (IgG)- like (Fig. 3A) and non-IgG-like [31]. BiTEs were synthetized for the first time in 1995, six years after the generation of the first CAR structure [32]. BiTEs are non-IgG-like subtypes of BsAbs, consisting of two antigen recognition domains (single-chain variable fragments—scFv) connected via a linker (Fig. 3B). While BiTEs are a particular form of BsAbs, there are more than 100 other formats, each of which has its own advantages and disadvantages.

A review of some of these new generation T cell recruiting antibodies was published by Zheng et al. [33]. The potency of T cell engagers is influenced by multiple factors, including the molecular target selected, the valency of the antibody or the spacing and spatial configuration of the antigen-binding domain. Studies have demonstrated that IgG-[L]-scFv-based T cell engagers exhibit a higher potency than those based on BiTEs or IgG heterodimer structure [34].

BiTEs activate anti-tumor cytotoxic and cytolytic mechanisms by indirectly linking MM cells with the T cells of patients. CD3, a T cell receptor (TCR) subunit, is the main antigen targeting T host cells. All BiTE trials for MM are based on CD3-mediated, MHC-independent T cell activation. Through these antibodies, an indirect immunological synapse is formed, leading to the same killing mechanisms described in the introduction of CARs.

### CAR and BiTE targets against MM cells in clinical trial phase

**Fig. 4:**
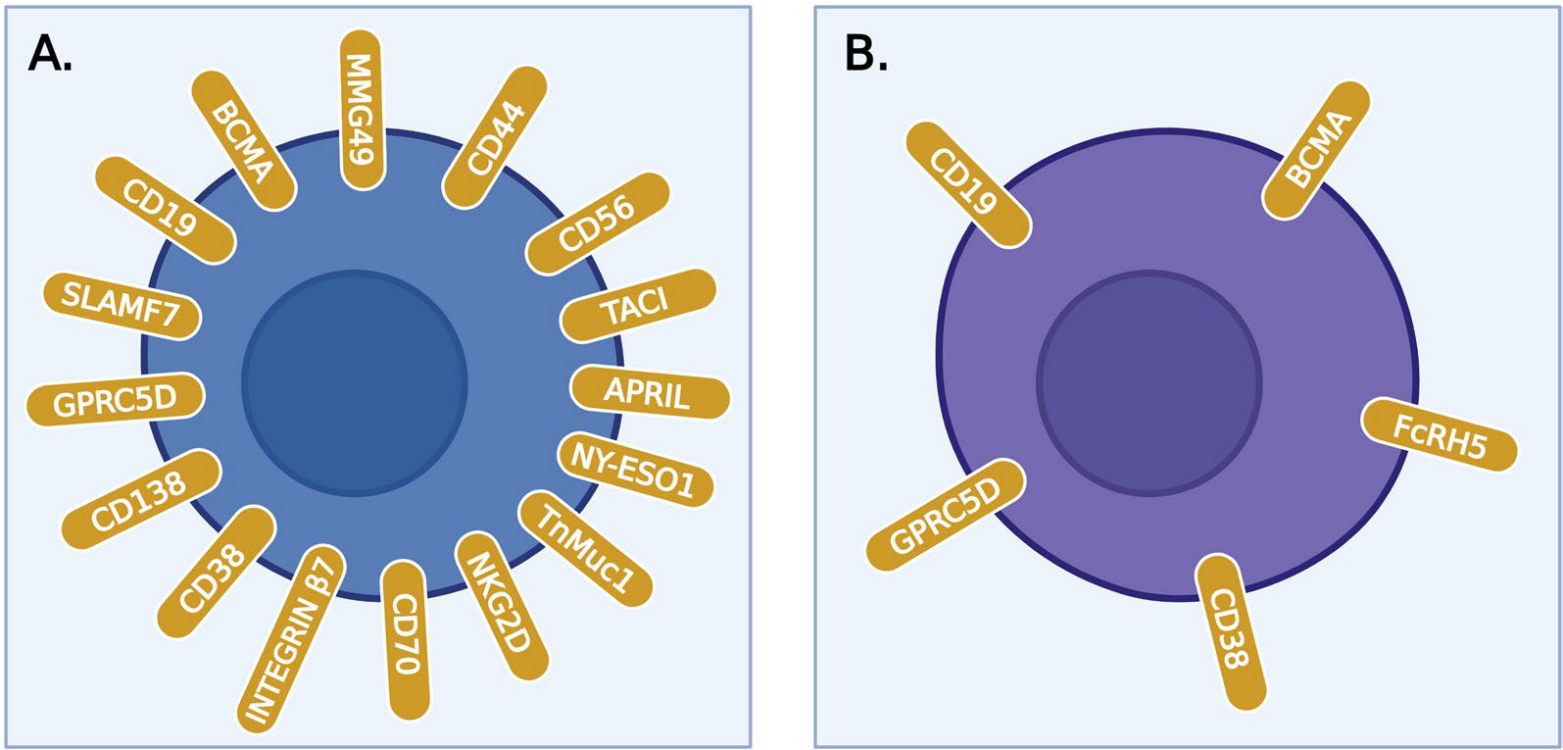
Surface antigens found on MM cells, studied in clinical trials. **A** CAR clinical trials; **B** BiTE clinical trials

A selection of adequate targets represents a challenge for scientists as it requires identification of epitopes present at high concentrations on MM cells, but not expressed in non-malignant hematopoietic cells or other tissues. We identified 3257 MM trials, of which 130 are CAR trials and 36 BiTE trials. The CAR and BiTE trials are detailed in Tables 1 and 2, respectively. Figure 4 illustrates all surface antigens currently being studied in these adoptive T cell therapy clinical trials.

**Table 1:** Car clinical trials

Trial							Outcomes		Adverse effects	
Target	Agent	Trial ID + references	Status	Phase	Study population	Enrollment (N)	ORR (%)	Median PFS (months)	Grade ≥ 3 CRS (%)	Grade 3 NTX (%)
BCMA	CAR-BCMA	NCT02215967 [37]	Completed	1	RRMM	24	81	7.75	25	4
BCMA	CAR T-BCMA (UPenn)	NCT02546167 [46]	Completed	1	HRMM	25	48	2.7	32	12
BCMA	BCMA CAR	NCT04650724 [109]	Completed	1	RRMM (extramedullary)	3 (data published for 1 patient)	100	3	0	0
BCMA	Idecabtagene vicleucel (bb2121)	NCT02658929 (CRB-401) [110]	Active, not recruiting	1	RRMM	67	76	8.8	6	3
BCMA	Idecabtagene vicleucel (bb2121)	NCT04196491 (KarMMa-4) [111]	Recruiting	1	NDMM, HRMM	60	76	8.8	6	3
BCMA	bb21217	NCT03274219 (CRB-402) [112]	Active, not recruiting	1	RRMM	72	55	11.9	4	6
BCMA	Zevorcabtagene autoleucel (CT053)	NCT03975907 (LUMMICAR-1) [113]	Recruiting	1	RRMM	62	87.5	18.8	6	3
BCMA	Zevorcabtagene autoleucel (CT053)	NCT03302403 [114]	Active, not recruiting	1	RRMM	18	87.5	Not yet reached	0	4
BCMA	Zevorcabtagene autoleucel (CT053)	NCT03716856 [114]	Active, not recruiting	1	RRMM	11	87.5	Not yet reached	0	4
BCMA	Zevorcabtagene autoleucel (CT053)	NCT03380039 [114]	active, not recruiting	1	RRMM	6	87.5	Not yet reached	0	4
BCMA	C-CAR088	NCT04322292 [115]	Recruiting	1	RRMM	10	95.2	Not yet reached	5	0
BCMA	C-CAR088	NCT03815383 [115]	Unknown	1	RRMM	12	95.2	Not yet reached	5	0
BCMA	C-CAR088	NCT03751293 [115]	Unknown	1	RRMM	10	95.2	Not yet reached	5	0
BCMA	C-CAR088	NCT04295018 [115]	Unknown	1	RRMM	10	95.2	Not yet reached	5	0
BCMA	BCMA nanobody CARs	NCT03661554 [116]	Unknown	1	RRMM	15	88.2	12.1	2.9	0
BCMA	FCARH143 (53)	NCT03338972	Active, not recruiting	1	RRMM	28	100	Not yet reached	6	0
BCMA	HRAIN Biotechnology	NCT03093168 [117]	Unknown	1	RRMM	10	86	Not yet reached	0	0
BCMA	IM21 CAR T	NCT04537442	Recruiting	1	RRMM, age ≥ 60	10	NA	NA	NA	NA
BCMA	IM21 CAR T	NCT03711864	Recruiting	1	RRMM	15	NA	NA	NA	NA
BCMA	BCMA-specific CAR T + gamma-secretase inhibitor LY3039478	NCT03502577 [55]	Suspended	1	RRMM	18	NA	NA	NA	NA
BCMA	KITE-585	NCT03318861	Active, not recruiting	1	RRMM	17	7	1	21	21
BCMA	CC-98633	NCT04394650	Recruiting	1	RRMM	80	NA	NA	NA	NA
BCMA	Human BCMA-targeted T Cells	NCT04003168	Recruiting	1	RRMM	18	NA	NA	NA	NA
BCMA	BCMA CAR T cells	NCT05150522	Recruiting	1	RRMM	10	NA	NA	NA	NA
BCMA	anti-BCMA CAR T (carbiogene)	NCT04637269	Recruiting	1	RRMM	16	NA	NA	NA	NA
BCMA	BCMA CAR T + immune inhibitors	NCT03943472	Recruiting	1	RRMM	10	NA	NA	NA	NA
BCMA	BCMA-UCART	NCT03752541	Recruiting	1	RRMM	20	NA	NA	NA	NA
BCMA	CXCR4-modified anti-BCMA CAR T cells	NCT04727008	Not yet recruiting	1	RRMM	12	NA	NA	NA	NA
BCMA	BCMA-targeted CAR T cells	NCT04670055	Not yet recruiting	1	RRMM	50	NA	NA	NA	NA
BCMA	BCMA-CAR T cells	NCT04186052	Unknown	1	RRMM	10	NA	NA	NA	NA
BCMA	NEXI-002	NCT04505813	Recruiting	1	RRMM	22	NA	NA	NA	NA
BCMA	BCMA CAR T	NCT03559764	Unknown	1	RRMM	20	NA	NA	NA	NA
BCMA	BCMA CAR T	NCT04626752	Recruiting	1	RRMM	50	NA	NA	NA	NA
BCMA	CBG-002	NCT04706936	Recruiting	1	RRMM	25	NA	NA	NA	NA
BCMA	FHVH-BCMA-T	NCT03602612 [58]	Active, not recruiting	1	RRMM	35	NA	NA	NA	NA
BCMA	HB10101	NCT04720313	Recruiting	1	RRMM	48	NA	NA	NA	NA
BCMA	CAR T-ddBCMA	NCT04155749	Recruiting	1	RRMM	65	NA	NA	NA	NA
BCMA	BCMA CAR T	NCT03322735	Unknown	1	RRMM	10	NA	NA	NA	NA
BCMA	CAR T re-treatment	NCT03672253	Unknown	1	RRMM	20	NA	NA	NA	NA
BCMA	CT103A (IBI326)	NCT05066646 (FUMANBA-1)	Recruiting	1	RRMM	132	94.9	Not yet reached	3	0
BCMA	CT103A (IBI326)	NCT05201118	Not yet recruiting	1	RRMM (extramedullary)	20	NA	NA	NA	NA
BCMA	CT103A (IBI326)	NCT05181501	Not yet recruiting	1	NDMM, HRMM	20	NA	NA	NA	NA
BCMA	LCAR-BCX	NCT04601935	Recruiting	1	RRMM	34	NA	NA	NA	NA
BCMA	PHE885	NCT04318327 [118]	Recruiting	1	RRMM	48	NA	NA	NA	0
BCMA	JWCAR129	NCT04677452	Recruiting	1	RRMM	24	NA	NA	NA	NA
BCMA	LCAR-B4822M	NCT03674463	Unknown	1	RRMM	10	NA	NA	NA	NA
BCMA	ALLO-715 CAR + ALLO-647 (anti-CD52 mAb)	NCT04093596 (UNIVERSAL)	Active, not recruiting	1	RRMM	132	NA	NA	NA	NA
BCMA	BCMA nanobody CAR T cells	NCT03664661	Recruiting	1	RRMM	15	NA	NA	NA	NA
BCMA	P-BCMA-ALLO1	NCT04960579 [40–42]	Not yet recruiting	1	RRMM	40	NA	NA	NA	NA
BCMA	MCARH171 + lenalidomide	NCT03070327 [119]	Active, not recruiting	1	RRMM	20	64	NA	20	0
BCMA and/or CD19	anti-BCMA-CD19 CAR T	NCT03767725	Unknown	1	RRMM	10	NA	NA	NA	NA
BCMA and/or CD19	Autologous BCMA CAR T cells and CD19 CAR T cells	NCT04194931	Unknown	1	RRMM	20	NA	NA	NA	NA
BCMA and CD19	CAR T-BCMA + huCART19	NCT03549442	Active, not recruiting	1	HRMM	40`	NA	NA	NA	NA
BCMA or CD19	CD19/BCMA-targeted CAR T cells + dasatinib	NCT04603872	Recruiting	1	RRMM	120	NA	NA	NA	NA
CD138 or integrin β7 or SLAMF7 or CD38 or BCMA	CAR T therapy	NCT03778346	Recruiting	1	RRMM	30	NA	NA	NA	NA
BCMA x CD19	BCMA/CD19 dual-target CAR T cell	NCT04412889	Not yet recruiting	1	RRMM	18	NA	NA	NA	NA
BCMA x CD19	GC012F	NCT04236011 [120]	Recruiting	1	RRMM	33	94.7	Not yet reached	10.5	0
BCMA x CD19	GC012F	NCT04617704	Active, not recruiting	1	NDMM, HRMM	15	NA	NA	NA	NA
BCMA x CD19	GC012F	NCT04182581 [120]	Unknown	1	RRMM	18	94.7	Not yet reached	10.5	0
BCMA x CD19	BCMA/CD19 CAR T	NCT04795882	Not yet recruiting	1	RRMM	24	NA	NA	NA	NA
BCMA x CD19	BCMA-CD19 cCAR T cells	NCT04162353 [52]	Recruiting	1	RRMM	12	95	8	NA	NA
BCMA x CD19	Anti-CD19/BCMA CAR T cells	NCT03706547	Recruiting	1	RRMM	20	NA	NA	NA	NA
BCMA x TACI	TriPRIL CAR T Cells	NCT05020444	Recruiting	1	RRMM	18	NA	NA	NA	NA
BCMA x TACI	APRIL CAR T cells	NCT04657861	Not yet recruiting	1	RRMM	36	NA	NA	NA	NA
BCMA x TACI	AUTO2	NCT03287804 [121]	Terminated	1	RRMM	12	43	Not published	0	0
BCMA x SLAMF7	CS1/BCMA Bispecific CAR	NCT04662099	Recruiting	1	RRMM	24	NA	NA	NA	NA
BCMA x SLAMF7	BCMA-CS1 cCAR T cells	NCT04156269	Unknown	1	RRMM	12	NA	NA	NA	NA
BCMA	Idecabtagene vicleucel (bb2121)	NCT04855136 (KarMMa-7)	Recruiting	1/2	RRMM	415	NA	NA	NA	NA
BCMA	Ciltacabtagene autoleucel (JNJ-68284528, LCAR-B38M)	NCT03548207 (CARTITUDE-1) [122]	Active, not recruiting	1/2	RRMM	126	98	Not yet reached	4	10
BCMA	Ciltacabtagene autoleucel (JNJ-68284528, LCAR-B38M)	NCT03090659 (LEGEND-2) [123]	Active, not recruiting	1/2	RRMM	100	88	19.9	41	0
BCMA	Orvacabtagene autoleucel (JCARH125)	NCT03430011 (EVOLVE) [124]	Active, not recruiting	1/2	RRMM	169	91	Not yet reached	2	4
BCMA	Zevorcabtagene autoleucel (CT053)	NCT03915184 (LUMMICAR-2) [125]	Recruiting	1/2	RRMM	105	100	Not yet reached	0	0
BCMA	P-BCMA-101	NCT03288493 (PRIME) [40–42]	Active, not recruiting	1/2	RRMM	220	57	Not yet reached	2	0
BCMA	DESCARTES 08	NCT04816526	Recruiting	1/2	NDMM, HRMM	30	NA	NA	NA	NA
BCMA	DESCARTES 11	NCT03994705	Recruiting	1/2	RRMM	18	NA	NA	NA	NA
BCMA	ARI0002h	NCT04309981 [126]	Recruiting	1/2	RRMM	36	NA	NA	NA	NA
BCMA	PBCAR269A + nirogacestat (gamma-secretase inhibitor)	NCT04171843	Recruiting	1/2	RRMM	48	NA	NA	NA	NA
BCMA	BCMA CAR T cells	NCT04272151	Recruiting	1/2	RRMM	40	NA	NA	NA	NA
BCMA	BCMA-targeted prime CAR T cells	NCT04776330	Recruiting	1/2	RRMM	80	NA	NA	NA	NA
BCMA	BCMA CAR T cells	NCT04271644	Recruiting	1/2	RRMM	80	NA	NA	NA	NA
BCMA	Anti-BCMA-CAR Transduced T cells	NCT02954445	Unknown	1/2	RRMM	45	NA	NA	NA	NA
BCMA	SENL-B19	NCT03312205 [127]	Recruiting	1/2	RRMM	50	NA	NA	19	NA
BCMA	ALLO-605 CAR + ALLO-647 (anti-CD52 mAb)	NCT05000450	Active, not recruiting	1/2	RRMM	136	NA	NA	NA	NA
BCMA	spCART-269	NCT04500431	Recruiting	1/2	RRMM	10	NA	NA	NA	NA
CD138 and BCMA and CD19	CAR T-138/BCMA/19/MORE	NCT03196414 [128]	Recruiting	1/2	RRMM	10	80	NA	NA	0
NY-ESO-1 + CD38 + BCMA + CD19	MULTI-target CAR T cell	NCT03638206	Recruiting	1/2	RRMM	73	NA	NA	NA	NA
BCMA, CD38, CD138, CD56	MULTI-CAR T	NCT03271632	Recruiting	1/2	RRMM	20	NA	NA	NA	NA
BCMA x CD38	Dual Specificity CD38 and BCMA CAR T	NCT03767751	Recruiting	1/2	RRMM	80	NA	NA	NA	NA
BCMA x CD19	GC012F	NCT04935580	Recruiting	1/2	NDMM, HRMM	20	NA	NA	NA	NA
BCMA x CD19	CD19-BCMA CAR T cells	NCT04714827	Recruiting	1/2	RRMM	24	NA	NA	NA	NA
BCMA and CD19	anti-CD19 and anti-BCMA CAR + auto-HSCT	NCT03455972	Recruiting	1/2	NDMM, HRMM	15	NA	NA	NA	NA
BCMA	Idecabtagene vicleucel (bb2121)	NCT03361748 (KarMMa) [129]	Active, not recruiting	2	RRMM	149	73	8.8	5	3
BCMA	Idecabtagene vicleucel (bb2121)	NCT03601078 (KarMMa-2)	Recruiting	2	NDMM, RRMM	181	NA	NA	NA	NA
BCMA	Idecabtagene vicleucel (bb2121)	NCT05032820	Not yet recruiting	2	RRMM	40	NA	NA	NA	NA
BCMA	Ciltacabtagene autoleucel (JNJ-68284528, LCAR-B38M)	NCT04133636 (CARTITUDE-2) [130]	Recruiting	2	NDMM, RRMM	160	95	Not yet reached	10	0
BCMA	Ciltacabtagene autoleucel (JNJ-68284528, LCAR-B38M)	NCT03758417 (CARTIFAN-1)	Recruiting	2	RRMM	130	NA	NA	NA	NA
BCMA	DESCARTES 08	NCT03448978	Recruiting	2	RRMM	30	NA	NA	NA	NA
BCMA	DESCARTES 11	NCT04436029	Recruiting	2	NDMM, HRMM	30	NA	NA	NA	NA
BCMA	PHE885	NCT05172596	Not yet recruiting	2	RRMM	100	NA	NA	NA	NA
BCMA	BMCA-targeted CAR T	NCT03931421	Recruiting	2	RRMM	30	NA	NA	NA	NA
BCMA x PD-1	BCMA-PD-1-CAR T Cell	NCT04162119	Recruiting	2	RRMM	30	NA	NA	NA	NA
BCMA	Idecabtagene vicleucel (BB2121)	NCT03651128 (KarMMa-3) [131]	Recruiting	3	RRMM	381	NA	NA	NA	NA
BCMA	Ciltacabtagene autoleucel (JNJ-68284528, LCAR-B38M)	NCT04181827 (CARTITUDE-4)	Active, not recruiting	3	RRMM	419	NA	NA	NA	NA
BCMA	Ciltacabtagene autoleucel (JNJ-68284528, LCAR-B38M)	NCT04923893 (CARTITUDE-5)	Recruiting	3	ASCT ineligible MM patients	650	NA	NA	NA	NA
BCMA	BCMA-directed CAR T cells + lenalidomide	NCT04287660	Recruiting	3	NDMM	20	NA	NA	NA	NA
BCMA	Ciltacabtagene autoleucel (JNJ-68284528, LCAR-B38M)	NCT05201781	Not yet recruiting	4	Janssen-sponsored cilta-cel patients	228	NA	NA	NA	NA
CD19	CTL019	NCT02135406 [91]	Completed	1	RRMM	10	80	Not published	0	0
CD19	CAR T-19 cells	NCT02794246	Terminated	2	RRMM	6	NA	NA	NA	NA
SLAMF7	CS1-CAR T Therapy	NCT03710421	Recruiting	1	RRMM	30	NA	NA	NA	NA
SLAMF7	UCARTCS1A	NCT04142619 (MELANI-01)	Recruiting	1	RRMM	18	NA	NA	NA	NA
SMLAF7	CS1-targeted CAR T cells	NCT04541368	Not yet recruiting	1	RRMM	50	NA	NA	NA	NA
SLAMF7	Anti-SLAMF7 CAR T cells	NCT03958656	Completed	1	RRMM	13	NA	NA	NA	NA
SLAMF7	SLAMF7 CAR T	NCT04499339 (CARAMBA-1)	Recruiting	1/2	RRMM	38	NA	NA	NA	NA
GPRC5D	CAR-GPRC5D	NCT05219721	Not yet recruiting	1	RRMM	18	NA	NA	NA	NA
GPRC5D	GPRC5D-CAR T	NCT05016778	Recruiting	1	RRMM	15	NA	NA	NA	NA
GPRC5D	MCARH109	NCT04555551	Active, not recruiting	1	RRMM	17	83	Not yet published	8	0
CD138	CAR138 T Cells	NCT03672318	Recruiting	1	RRMM	33	NA	NA	NA	NA
CD138	CAR T-138	NCT01886976 [95]	Recruiting	1/2	RRMM	10	80	NA	0	0
CD38	CAR2 Anti-CD38 A2 CAR T Cells	NCT03464916	Active, not recruiting	1	RRMM	72	NA	NA	NA	NA
CD70	CD70 CAR	NCT04662294	Recruiting	1	RRMM	108	NA	NA	NA	NA
NKG2D	CM-CS1 T cell infusion	NCT02203825 [85]	Completed	1	RRMM	12	NA	NA	0	0
NKG2D	NKR-2 (CYAD-01)	NCT03018405 (THINK) [86]	Recruiting	1/2	RRMM	146	46	NA	18.8	0
TnMuc1	CAR T-TnMUC1	NCT04025216 [94]	Recruiting	1	RRMM	112	NA	NA	0	0
CD44v6	MLM-CAR44.1 T cells	NCT04097301	Terminated	1/2	RRMM	48	NA	NA	NA	NA
MMG49	OPC-415	NCT04649073	Recruiting	1/2	RRMM	49	NA	NA	NA	NA
PD-1 x not published	Novel CAR T	NCT04191941	Unknown	1	RRMM	9	NA	NA	NA	NA
no data published	C-4–29 Dual-target CAR T cells	NCT04861480	Recruiting	NA	RRMM	18	NA	NA	NA	NA

*RRMM* = relapsed/refractory MM, *NDMM* = newly diagnosed MM, *HRMM* = high-risk MM, *ORR* = overall response rate, *PFS* = progression-free survival, *CRS* = cytokine release syndrome, *NTX* = neurotoxicity

**Table 2:** BiTE clinical trials

Trial						Structure		Outcomes		Adverse effects	
Agent	Trial ID + references	Status	Phase	Study population	Enrollment (N)	MM Cell target	T Cell Ligand	ORR (%)	MEDIAN PFS (months)	Grade ≥ 3 CRS (%)	Grade 3 NTX (%)
AMG 420 (pacanalotamab, BI 836,909)	NCT03836053	Active, not recruiting	1	RRMM	47	BCMA	CD3	NA	NA	NA	NA
AMG 420 (pacanalotamab, BI 836,909)	NCT02514239 [132]	Completed	1	RRMM	42	BCMA	CD3	70	23.5	2	4
AMG 701 (pavurutamab)	NCT04998747 (ProxiMMity-1)	Not yet recruiting	1	RRMM	47	BCMA	CD3	NA	NA	NA	NA
elranatamab (PF-06863135)	NCT03269136 (MagnetisMM-1) [133]	Active, not recruiting	1	RRMM	90	BCMA	CD3	75	Not published	0	0
elranatamab (PF-06863135)	NCT04798586 (MagnetisMM-2)	Active, not recruiting	1	RRMM	4	BCMA	CD3	NA	NA	NA	NA
teclistamab (JNJ-64007957)	NCT03145181 (MajesTEC-1) [134]	Recruiting	1	RRMM	204	BCMA	CD3	67	Not published	0	3
teclistamab (JNJ-64007957) + anticancer drugs	NCT04722146	Recruiting	1	RRMM	140	BCMA	CD3	NA	NA	NA	NA
teclistamab and talquetamab	NCT04586426 (RedirecTT-1)	Recruiting	1	RRMM	56	BCMA and GPRC5D	CD3	NA	NA	NA	NA
teclistamab (or talquetamab) + daratumumab	NCT04108195 (TRIMM-2) [135]	Recruiting	1	RRMM	200	BCMA or GPRC5D	CD3	78	Not published	0	0
alnuctamab (CC-93269, EM901)	NCT03486067 [136]	Recruiting	1	RRMM	175	BCMA	CD3	83	Not published	5	0
TNB-383B	NCT03933735 [137]	Recruiting	1	RRMM	169	BCMA	CD3	52	Not published	0	0
WVT078	NCT04123418	Recruiting	1	RRMM	90	BCMA	CD3	NA	NA	NA	NA
REGN5458	NCT05137054	Not yet recruiting	1	RRMM	256	BCMA	CD3	NA	NA	NA	NA
teclistamab (JNJ-64007957)	NCT04696809	Recruiting	1/2	RRMM	33	BCMA	CD3	NA	NA	NA	NA
AMG 701 (pavurutamab) + pomalidomide	NCT03287908 [138]	Recruiting	1/2	RRMM	408	BCMA	CD3	82	Not published	7	0
elranatamab (PF-06863135) + gamma-secretase inhibitor	NCT05090566 (MagnetisMM-4)	Recruiting	1/2	RRMM	65	BCMA	CD3	NA	NA	NA	NA
elranatamab (PF-06863135)	NCT05014412 (MagnetisMM-9)	Recruiting	1/2	RRMM	76	BCMA	CD3	NA	NA	NA	NA
REGN5458	NCT03761108 [139]	Recruiting	1/2	RRMM	292	BCMA	CD3	60	Not published	0	0
REGN5459	NCT04083534	Active, not recruiting	1/2	RRMM	43	BCMA	CD3	NA	NA	NA	NA
EMB-06	NCT04735575	recruiting	1/2	RRMM	66	BCMA	CD3	NA	NA	NA	NA
teclistamab (JNJ-64007957)	NCT04557098 (MajesTEC-1) [140]	Recruiting	2	RRMM	192	BCMA	CD3	65	Not yet reached	0	0
elranatamab (PF-06863135)	NCT04649359 (MagnetisMM-3)	Active, not recruiting	2	RRMM	180	BCMA	CD3	NA	NA	NA	NA
elranatamab (PF-06863135)	NCT05228470 (MagnetisMM-8)	Recruiting	2	RRMM	36	BCMA	CD3	NA	NA	NA	NA
talquetamab (JNJ-64407564)	NCT03399799 [75]	Recruiting	1	RRMM	260	GPRC5D	CD3	63	Not yet published	4	0
talquetamab (JNJ-64407564) + anticancer drugs	NCT05050097	Recruiting	1	RRMM	176	GPRC5D	CD3	NA	NA	NA	NA
talquetamab (JNJ-64407564)	NCT04773522	Recruiting	1	RRMM	9	GPRC5D	CD3	NA	NA	NA	NA
talquetamab (JNJ-64407564)	NCT04634552	Recruiting	2	RRMM	320	GPRC5D	CD3	NA	NA	NA	NA
blinatumomab	NCT03173430	Terminated	1	RRMM	6	CD19	CD3	NA	NA	NA	NA
AMG 424	NCT03445663	Terminated	1	RRMM	27	CD38	CD3	NA	NA	NA	NA
Y150	NCT05011097	Recruiting	1	RRMM	75	CD38	CD3	NA	NA	NA	NA
GBR1342 (ISB 1342)	NCT03309111	Recruiting	1	RRMM	197	CD38	CD3	NA	NA	NA	NA
cevostamab (BFCR4350A, RG6160)	NCT04910568 (CAMMA 1)	Recruiting	1	RRMM	120	FcRH5	CD3	NA	NA	NA	NA
cevostamab (BFCR4350A, RG6160)	NCT03275103 (GO39775) [141]	Recruiting	1	RRMM	300	FcRH5	CD3	52	Not yet reached	2	0

*RRMM* = relapsed/refractory MM, *ORR* = overall response rate, *PFS* = progression-free survival, *CRS* = cytokine release syndrome, *NTX* = neurotoxicity

## Potential therapeutic targets for MM

### BCMA CAR

A B cell maturation antigen (BCMA) (CD269 or TNFRSF17) is a non-tyrosine kinase receptor surface glycoprotein, belonging to the tumor necrosis factor (TNF) receptor superfamily. It is expressed in plasma cells, but not memory B and naïve B cells, nor in hematopoietic stem cells or T lymphocytes. Rare BCMA-positive cells can be identified in normal tissue cells such as lymph nodes, the spleen, the lungs and the stomach [35], but with limited expression. BCMA is highly expressed on the surface of MM cells [36].

The first BCMA-targeted CAR trial was developed in 2016 (NCT02215967). The therapy has shown great success with an overall response rate (ORR) of 81% in this phase 1 trial [37]. Furthermore, a meta-analysis including 22 CAR trials demonstrated an ORR of 85.2% with a median progression-free survival (PFS) of 14 months and an overall survival (OS) of 24 months [38]. Consequently, novel BCMA CARs were engineered, and old CARs were fine-tuned to improve safety and efficacy. Currently, 107 out of 130 CAR trials in MM target BCMA. Novel manufacturing techniques for CARs are currently available. BCMA-targeting mRNA CARs were generated and are now clinically evaluated (NCT03994705, NCT04436029) [39].

Safety and efficacy of BCMA CARs manufactured by a transposon-based system is also tested (NCT04960579, NCT03288493) [40–42]. These products favor the development of memory stem cells. Thus, the persistence of CARs and treatment efficacy could be increased. Three possible hypotheses were found to play a role in the development of memory stem cell phenotypes when using a transposon-based expression of CARs: (1) the effects of the 4-1BB domain, which promotes outgrowth of memory stem cells; (2) different cytokines added to the medium could also play a role in the outgrowth of different subpopulations; and (3) the vector itself, used for gene transfer, could influence the ratio of subpopulations. In spite of the lack of clarity regarding its mechanism, studies have demonstrated that transposon-based CARs are superior to lentiviral-transduced ones regarding memory stem cell phenotype development [43].

It is also assumed that T cell subpopulation ratios should be adjusted during the manufacturing process to enhance memory stem cell persistency [44]. Thus, the CD4 + /CD8 + ratio is adjusted to 1:1 before CARs’ gene transfer in case of JCARH125 (NCT03430011) or after gene transfer in case of FCARH143 product (NCT03338972) [45].

Current debate exists regarding the questions of whether a BCMA expression threshold should be defined as an inclusion criterion in BCMA CAR studies because contradictory results have been published regarding this aspect. Cohen et al. showed no correlation between BCMA expression and response rate (NCT02546167) [46] as opposed to the FCARH143 trial (NCT03338972) [47]. Van de Donk et al. reviewed hypotheses regarding this controversy. Possible determinants could be differences in assays (flow cytometry versus immunohistochemistry) used to quantify BCMA expression or the effects of soluble BCMA (sBCMA) formed by shedding of MM cell membranes [48]. Furthermore, some authors relate that sBCMA plasma levels could be a valid biomarker in response assessment in the future [49].

CAR trials mostly enroll relapsed/refractory MM patients. However, a few trials for newly diagnosed patients were launched (NCT04196491, NCT04816526, NCT04436029). The majority of BCMA CAR trials study BCMA alone, but dual CAR products are also available, combining BCMA and CD19 (NCT04236011, NCT04162353) [50–52], BCMA and SLAMF7 (NCT04662099, NCT04156269) or BCMA and CD38 (NCT03767751). Administering dual CARs can be achieved by two approaches: either by coinfusion of two distinct CARs or by infusion of a single CAR product, expressing both antigens [53]. A trial of a multi-CAR product, which expresses several antigen-binding domains directed toward BCMA, CD38, CD138 and CD56, was launched recently (NCT03271632).

Other ways of improving efficacy of CARs are evaluated, such as associating BCMA CARs with tyrosine kinase inhibitors (NCT04603872), immune modulators (NCT04287660, NCT03070327) or other non-specified immune inhibitors (NCT03943472), as well as by associating BCMA CARs with tyrosine kinase inhibitors (NCT04603872), immune modulators (NCT04287660, NCT03070327) or other non-specified immune inhibitors (NCT03943472) or the concomitant secretion of inhibitory binders for PD-1. Clinical data show that BCMA is directly cleaved by gamma-secretase, a membrane-bound protease. Furthermore, it is assumed that the administration of gamma-secretase inhibitors increases BCMA expression [54]. Thus, BCMA CARs coadministered with gamma-secretase inhibitors are now being clinically evaluated (NCT03502577) [55].

Third-generation CARs, with two costimulatory moieties, have been shown to be clinically efficient with high response rates (NCT03196414) [56]. Clinical trials evaluating safety and efficacy of fourth-generation BCMA CARs secreting IL7/CCL9 (NCT03778346) or a mutant PD-1 ligand (NCT04162119) are ongoing as well.

Currently, one clinical trial is investigating the efficacy of BCMA CAR cells for MM patients refractory to previous CAR treatment (NCT03672253). CAR trials use preponderantly autologous T lymphocytes. However, one allogeneic healthy donor-derived BCMA CAR product, PBCAR269A, is also being evaluated, intending to generate an off-the-shelf drug (NCT04171843). An additional risk associated with the administration of allogeneic CARs is the development of a graft versus host disease (GvHD). In order to confer lymphodepletion resistance and reduced GvHD potential, Sommer et al. developed transcription activator-like effector nuclease (TALEN) gene-edited CAR T cells. A mimotope-based CD20 CAR off-switch was integrated into the construct. Consequently, effective CAR elimination was made possible when rituximab is administered [57]. BCMA CARs with nanobody antigen-binding domains are tested in Phase I trials (NCT03664661, NCT03602612, NCT03661554) [58]. Chemokine receptor CXCR4-modified BCMA CARs are being investigated as a new treatment option (NCT04727008) [59].

In order to reduce toxicities, trials evaluating BCMA CAR engineered with a truncated epidermal growth factor receptor (EGFRt) suicide gene system [60] have been launched (NCT03070327, NCT03093168). Some MM patients develop complications, such as amyloid light chain (AL) amyloidosis. A BCMA CAR clinical trial suggests that these patients could be treated with CAR (NCT04309981).

The first FDA-approved BCMA CAR product for MM is idecabtagene vicleucel (bb2121, ide-cel). The phase II clinical trial coordinated by San-Miguel et al. on 140 enrolled patients, out of which 128 received idecabtagene vicleucel, showed a 73% response rate and a 30% complete response. Cell kinetics analysis confirmed the presence of CARs in 69% of patients after 6 months and 36% after one year following infusion [61].

Ciltacabtagene autoleucel (cilta-cel), known as LCAR-B38M in China and JNJ 68,284,528 (JNJ 4528) in the USA, targets double epitopes of BCMA using two tandem VHH sequences. Double targeting makes possible the efficient depletion of low BCMA-expressing MM cells. Cilta-cel is an autologous therapy and one of the most studied BCMA CAR products. It has and is now being studied in the phase 1/2 CARTITUDE-1 (NCT03548207), LEGEND-2 (NCT03090659), phase 2 CARTITUDE-2 (NCT04133636), CARTIFAN-1 (NCT03758417), phase 3 CARTITUDE-4 (NCT04181827) and CARTITUDE-5 (NCT04923893). The efficacy of cilta-cel was compared to the standard PVd (pomalidomide, bortezomib, dexamethasone) regime in the CARTITUDE-4 trial. VRd (bortezomib, lenalidomide and dexamethasone) induction followed by cilta-cel was assessed in CARTITUDE-5. Based on the results of CARTITUDE-1, the FDA-approved cilta-cel in 28th February of 2022. A Phase 4 study is also ongoing, aiming to evaluate the long-term effects of cilta-cel (NCT05201781).

Several studies are testing its efficacy and safety profile in phase I CRB-401 (NCT02658929) and KarMMa-4 (NCT04196491); in phase 1/2 KarMMA-7 (NCT04855136); in phase 2 KarMMa (NCT03361748), KarMMa-2 (NCT03601078) and NCT05032820; and in phase 3 KarMMa-3 (NCT03651128). Derived from ide-cel, bb21217 uses the same CARs construct as bb2121; but a PI3K inhibitor (bb007) is added during ex vivo culturing, aiming both to enrich the memory-like T cell subpopulation and reduce T cell senescence (NCT03274219).

### BCMA BiTE

Similar to CAR trials, BiTE studies target predominantly BCMA as an MM cell antigen, representing 26 out of 35 current BiTE studies. All BiTEs currently investigated in MM clinical trials bind to T lymphocytes by a CD3 ligand. The first BiTE trial for MM investigated the safety and efficacy of AMG420 (pacanalotamab, BI 836,909). Currently, this trial (NCT02514239) is the first and only completed clinical BiTE study for MM. There were 42 patients enrolled. 70% responded to treatment with a median PFS of 23.5 months. Another ongoing phase 1 AMG420 study is evaluating intermittent dosing of the drug (NCT03836053). Based on AMG420, a novel BiTE, AMG701 (NCT04998747) was developed. AMG701 has been supplemented by an additional single-chain crystallizable fragment (scFv), which allows delayed renal clearance and extended half-life. Thus, while AMG420 requires daily administering, AMG701 allows once-weekly dosing. The AMG701 trial enrollment was apparently halted by adverse events. No information has been found indicating that the trial has been restarted.

BiTEs originally contained tscFv antigen-binding domains. However, IgG-like BiTEs and BiTEs with what and are now clinically evaluated. With the advance in technologies for bispecific antibodies and antibody discovery, additional formats, including IgG—alnuctamab (NCT03486067) or heavy-chain-only anti-BCMA moieties such as TNB-383B (NCT03933735), have also been engineered and appear to be entering the clinic at an accelerated rate. It is unique to BiTEs that they allow an immune synapse to form more quickly and effectively than other formats requiring an even more strict requirement of epitopes for high potency.

Similar to CAR, combining BiTE with anticancer drugs such as lenalidomide, pomalidomide, daratumumab or bortezomib is being investigated (NCT04722146). The MagnetisMM-5 (NCT05020236) study combines elranatamab with the monoclonal antibody daratumumab. Teclistamab is also tested as associated with daratumumab (NCT04108195) with the aim of improving treatment efficacy.

Soluble BCMA released by the action of gamma-secretase can hinder BiTE pharmacological effects [62]]. Thus, the concomitant administration of elranatamab with gamma-secretase inhibitors is being investigated (NCT05090566). Thus, to prevent antigen escape, BiTEs targeting two different antigens—BCMA and GPRC5D—can be concomitantly administered (NCT04586426).

Elranatamab (PF-06863135) is currently being examined in 7 trials (MagnetisMM 1, 2, 4, 5, 8 and 9), both as a single agent and in combination. Teclistamab is another extensively studied T cell receptor, presenting an IgG format. It is being investigated in 7 trials under the names MajesTEC-1, RedirecTT-1, TRIMM-2, MajesTEC-3. Teclistamab could be the first BCMA-targeted T cell engager approved by the FDA.

## NON-BCMA antigen targets

Apart from BCMA, the non-BCMA antigen targets, normal tissue distribution, constructions and potential critical aspects are presented in Table 3.

**Table 3:** Antigen expression, normal tissue distribution, constructions, and potential critical aspects

Target	Expression by myeloma cells	Expression by normal hematopoietic cells	Expression on other tissues	Clinical development of treatment options	Critical issues during development
CD38	Increased and uniform expression. Downregulated after Mab treatment	Myeloid and lymphoid cells, progenitor cells, NK and T cells, neutrophils and dendritic cells	Epithelial cells (prostate), pancreatic islet cells, pulmonary cells, Purkinje neurons (cerebellum)	Mabs (FDA, EMA approved) CAR T (clinical trials)	Mab treatment well tolerated Expression on normal tissues can hamper the development of more potent therapies
BCMA	60–100%	Plasmocytes and plasmoblasts	Absent (controversial expression on basal ganglia (brain)	Antibody–drug conjugate (FDA, EMA approved) CAR T (FDA, EMA approved) Bispecific antibodies (BITE and IgG format)	Duration of response for some of the CAR T constructs Potential neurotoxicity
SLAMF7/CS1	Increased and uniform expression	NK, T and B cells, monocytes, macrophages, dendritic cells and plasmacytes	Absent	Mabs (FDA, EMA approved) CAR T (clinical trials)	Fratricide on NK cells
FcRL5	Expression on > 80% of patients	B cells and plasmocytes	Absent	Bispecific antibodies (BiTE format)	IV formulation
GPRC5D	High expression in > 60% patients	B cells and plasma cells	Epithelial cells of skin and of filiform papillae (tongue)	Bispecific antibodies (IgG format) and CAR T, both in clinical trials	Skin and nail toxicity, dysgeusia
CD138	High expression	Plasmocytes	Epithelial cells of GI tract, hepatocytes	CAR T (clinical trials)	Currently in early development
CD19	Weak expression	B cell lineage cells	Absent	CAR T (clinical trials)	Weak expression on tumor cells
CD56	High expression in 80% of patients	NK and T cells, monocytes	Neural expression	CAR T (clinical trials)	Currently in early development

### APRIL/TACI

Transmembrane activator, calcium modulator and cyclophilin ligand interactor (TACI) is a kinase receptor, closely related to BCMA. Both receptors play a role in B cell survival, but their expression levels are different at distinct stages of differentiation. BCMA and TACI have two main natural ligands: B cell activating factor (BAFF) and a proliferation-inducing ligand (APRIL). While BCMA is widely expressed in MM cells, TACI is usually present at lower levels and less frequently [63]. APRIL binds both BCMA and TACI with a high, nanomolar affinity. To circumvent the loss of BCMA antigen expression, a novel APRIL-based binding moiety was generated, in which a truncated form of APRIL was engineered as a tumor-targeting domain [64]. Novel, trimeric APRIL-based (TRIPRIL) CARs were also generated, aiming to enhance binding capacity [65]. These BCMA x TACI-targeting APRIL/TRIPRIL CAR products are now being clinically investigated (NCT04657861, NCT05020444, NCT03287804). We identified no APRIL/TACI-targeting BiTE products in the clinical trial phase.

### CD38

CD38 is a general lymphocyte receptor, highly expressed in MM cells. Anti-CD38 CARs induce the death of other immune cells (natural killer cells, monocytes, other B and T lymphocytes) as well; but progenitor cells are not killed, and their proliferation is not inhibited. To guarantee the safety of CD38-targeted cellular therapies, suicide genes can be inserted into the CAR construct [66]. Anti-CD38 CARs are currently under investigation in monotherapy (NCT03464916), in BCMAxCD38 combination (NCT03767751) or multitarget CAR settings, targeting several antigens (CD38, CD19, CD56, BCMA, CD138, NY-ESO1) at the same time (NCT03638206, NCT03271632). The efficacy of a fourth-generation anti-CD38 CAR is also being explored (NCT03778346). CD38 is a promising target in T cell engager constructs as well. AMG424 is the first-in-human CD38 antibody product, consisting of a hetero-Fc domain lacking the Fcз receptor, an anti-CD3 scFv domain and an anti-CD38 Fab fragment [67]. Although the phase 1 AMG424 trial (NCT03445663) was terminated, two other products, Y150 and GBR1342 [68], are currently being tested (NCT05011097, NCT03309111). Another novel product is Bi38-3, which proved effective in preclinical mice studies [69].

### SLAMF7

The signaling lymphocyte activation molecule (SLAM) family of receptors is exclusively found in hematopoietic cells. SLAMF7 (CS1—CND3 subset 1, CRACC, CD269) is a cell surface glycoprotein, whose enzymatic cleavage generates a soluble form [70]. Studies suggest that soluble SLAMF7 acts like a growth factor, causing MM cell proliferation [71]. CARAMBA-1 (NCT04499339) was the first-in-human clinical CAR trial targeting SLAMF7. CARAMBA-1 investigates CARs generated by the Sleeping Beauty transposon gene transfer system [72]. To enhance its safety profile, SLAMF7-CARs transduced with the iCasp9 suicide gene (activated by rimiducid) were also evaluated (NCT03958656), with no published results to date. Currently, 5 trials are studying SLAMF7-targeting CAR in monotherapy (NCT03710421, NCT04142619, NCT04541368, NCT03958656, NCT04499339). Trials with CARs targeting both BCMA and SLAMF7 are also ongoing (NCT04795882, NCT04156269). Fourth-generation anti-SLAMF7-CARs have been engineered and are now being clinically tested (NCT03778346). Of note, no SLAMF7-targeted BiTE is currently being investigated.

### GPRC5D

The G protein-coupled receptor, class C group 5 member D (GPRC5D), is a surface receptor expressed predominantly in hair follicles but can also be detected in MM cells. In preclinical studies, anti-GPRC5D CARs generated no alopecia or any skin damage and were deemed safe and efficient [73]. Currently, 3 GPRC5D-CAR T trials are ongoing (NCT05219721, NCT05016778, NCT04555551). MCARH109 is a promising CAR T product, obtaining an ORR of 83%, with an adequate safety profile. Increased GPRC5D expression is associated with poor prognosis [74]. In a phase 1 trial, Talquetamab (JNJ-64407564), a bispecific GPRC5D x CD3 IgG4 antibody, showed great potency with an ORR of 63% [75]. Currently, the effects of talquetamab are being studied in monotherapy (phase 1—NCT03399799, NCT04773522, phase 2—NCT04634552) or are associated with other anticancer drugs, such as carfilzomib, lenalidomide or daratumumab (NCT05050097). BCMA-targeting BiTE teclistamab concomitantly given with talquetamab is evaluated in the RedirecTT-1 (NCT04586426) and TRIMM-2 (NCT04108195) trials.

### FcRH5 (FcRL5, IRTA2, CD307)

The Fc receptor-like 5 (FcRL5/FcRH5/IRTA2/CD307) is a member of a receptor family known under a variety of names: immunoglobulin superfamily receptor translocation associated (IRTA), Fc receptor homolog (FcRH) or immunoglobulin superfamily-Fc receptor-gp42. The expression of FcRH5 starts in pre-B cells but reaches its peak only in mature B cells. Compared with normal plasma cells, MM and MGUS cells showed > threefold higher expression levels [76]. The development of CARs targeting FcRH5 is not yet underway. In contrast, the FcRH5-targeting BiTEs cevostamab (BFCR4350A, RG6160) has demonstrated promising results in the GO39775 trial (NCT03275103), achieving an ORR of roughly 52% in RRMM patients with only one (2%) patient presenting the grade 3 cytokine release syndrome. The efficiency of cevostamab plus pomalidomide and dexamethasone or cevostamab plus daratumumab and dexamethasone combinations is also being investigated in the CAMMA 1 study (NCT04910568).

### CD138

Syndecan-1 (CD138), a transmembrane proteoglycan, is a major extracellular matrix receptor that plays an important role in cell–cell and cell–matrix adhesion. Syndecan-1 is primarily expressed on the surface of mature epithelial cells but is also expressed by normal and malignant hematopoietic cells. The presence of a higher soluble CD138 concentration represents a negative prognostic factor in MM. O'Connell et al. found CD138 positivity in all 43 of the MM cases examined [77]. A total of 5 CAR trials targeting CD138 have been identified, mostly as a single target (NCT03196414, NCT01886976, NCT03672318, NCT03778346) but also as a multitarget construct (NCT03271632). Current data sustain the safety of anti-CD138 CARs therapy, as no cytokine release syndrome or neurotoxicity has been reported. The ORR is also promising, with 4 out of 5 patients (80%) achieving a significant reduction in tumor burden [78].

### CD19

The CD19 antigen is a cell surface glycoprotein expressed in B lymphocytes. Earlier studies assumed that MM cells were CD19 negative [79], while new studies suggest that some resistant MM progenitor cells are CD19 positive [80]. Thus, targeting CD19 could be a potential therapeutical approach to relapsed/refractory MM. For example, the use of anti-CD19 CARs, CTL019 (NCT02135406) in association with autologous hematopoietic stem cell transplantation in RRMM, was associated with an ORR of 80% and an improved duration of response as compared to previous transplantation in 2/10 patients [81].

Currently, only BCMA x CD19 double-targeting CAR trials are ongoing. Some of these products have reached impressive (94.7%/95%) overall response rates (NCT04182581, NCT04162353) [82, 83]. A CD19-targeting BiTE product, blinatumomab, has also been investigated, but the trial was terminated due to slow accrual (NCT03173430).

### NKG2D

NKG2D (natural-killer group 2, member D) is a cell surface receptor predominantly expressed in cytotoxic immune cells, such as NK cells or some T cell subsets, while playing a major role in tumor immunosurveillance. NKG2D is neither expressed in mature B cells nor B cell precursors. Tumoral cells usually upregulate stress-induced ligands, such as MIC-A, MIC-B and UL-16. Binding these ligands to NKG2D leads to the secretion of proinflammatory cytokines and to the activation/proliferation of cytotoxic cells, resulting in the elimination of tumoral cells [84]. The antigen-binding domain of NKG2D CARs is natural ligands, expressing the aforementioned immunoreceptor. Due to the presence of NKG2D's natural costimulatory domain, DAP10, NKG2D CARs do not require the addition of costimulatory regions in their construct. A disadvantage of these CARs is decreased T cell persistence. Therefore, multiple infusions and higher doses are required. Higher doses, however, have the same safety profile, with no reports of CRS or neurotoxicity [85]. Currently, the THINK trial (NCT03018405) is recruiting patients to test the efficacy of NKG2D CAR administration without prior chemotherapy or lymphodepletion. Based on the results received to date, anti-tumor effects on acute myeloid leukemia and myelodysplastic syndrome have been demonstrated. As of now, the results have not been reported for MM [86].

### CD56

The neural cell adhesion molecule (NCAM), known as CD56, is a membrane glycoprotein and member of the immunoglobulin superfamily. CD56 is an NK-cell surface marker not expressed in normal plasma cells, but 78% of MM cells showed CD56 positivity [87]. To counter antigen escape, a multitarget CAR was developed, targeting CD56 among others (NCT03271632).

#### Integrin-Beta7

The integrin-beta7 receptor subfamily is primarily expressed by leukocytes. High levels of expression in MM contribute to adhesion, migration, homing, invasion, drug resistance, as well as poor survival outcomes [88]. New generation CARs targeting MMG49, an epitope found in the beta7 chain's N-terminal region, were also developed. MMG49 is inaccessible in the resting integrin conformation but exposed in the active conformation. MMG49 reactivity was strong in MM cells due to increased expression and the constitutive activation of integrin-beta7, whereas MMG49 binding was barely detectable in other, normal cell types [89]. There is a full-length integrin-beta7 targeting CAR study (NCT03778346) currently underway, evaluating the receptor both in a monotarget and multitarget setting. Another study is investigating the safety and efficacy of MMG4- targeting CARs (NCT04649073).

#### CD44v6

CD44 is a ubiquitously expressed glycoprotein, cutting away the possibility of clinically significant anti-CD44 CARs. However, some CD44 isoforms, such as CD44v6, are absent in hematopoietic stem cells and barely found in normal cells, yet are highly expressed on the surface of MM cells [90]. We identified only one terminated CD44v6-targeted CAR trial for acute myeloid leukemia and MM (NCT04097301). In order to increase CAR safety, several suicide gene systems (thymidine kinase or inducible caspase 9) were evaluated, using CD44v6 CARs. No currently ongoing clinical trial was found.

#### NY-ESO-1

Cancer testis antigens (CTAs) are a set of tumor-associated antigens with limited expression in normal somatic tissues. However, they have been identified in a wide range of malignancies, including MM [91]. Such antigens are members of the GAGE family and NY-ESO-1, both expressed in one-third of MM patients; CTAG2, detected in half of MM patients; or the members of the MAGE family found in two-thirds of MM patients. Despite the fact that NY-ESO-1 is only detected in one-third of MM patients, it is the most immunogenic CTA. Because of the diverse expression of CTAs within MM cells, including many CTAs in a vaccine would be desirable. However, targeting NY-ESO-1 could have various negative consequences because it is also expressed in stem cells [92]. To increase its safety, only multitarget-CARs are now being clinically evaluated (NCT03638206).

#### TnMUC1

Membrane mucin 1 (MUC1) is a glycoprotein found in the majority of glandular epithelial cells as well as leukocytes. In some tumoral cells, aberrantly glycosylated proteins are present. An example is the Tn glycoform of MUC1, a tumor-associated neoantigen [93]. Therefore, TnMUC1-targeting CARs were engineered and are now clinically evaluated in a phase 1 study (NCT04025216). According to preliminary evidence, this CAR product can be safely provided to cancer patients [94].

#### PD-1

The expression of PD-L1 in tumor cells constitutes a major mechanism of immune escape by inhibition of T cell activation. In MM cells, PD-L1 expression was linked to higher proliferative potential and resistance to antimyeloma drugs [95]. Secretion of a PD-L1 blocker along with CARs expression is one strategy to improve CAR efficacy. We have identified one PD-L1-secreting CAR study in MM (NCT04191941).

#### CAR versus BiTE

Both CAR and BiTE offer benefits and drawbacks, which physicians should consider while deciding on the best treatment option. The high cost of CAR treatment is a key disadvantage to its widespread use. The overall cost of a CAR therapy can potentially reach 450.000 USD, depending upon numerous criteria (presence and severity of adverse effects, academic or non-academic location, pharmaceutical company) [96]. BiTE is anticipated to cost 72,000 USD, making it far more affordable to a public healthcare system [97]. Still, this price may be underestimated, as shown by Thielen et al., who demonstrated that in the case of relapsed/refractory acute lymphoblastic leukemia, the discounted costs for CAR were almost 150,000 EUR higher from a society point of view as compared with a healthcare point of view (552,679 EUR versus 409,553 EUR) and much higher than BiTE, estimated at 267,259 EUR [98].

Regarding the US Food and Drug Administration (FDA) approval, idecabtagene vicleucel is the only BCMA-targeting CAR currently approved. The first BiTE will most likely be approved by the FDA in 2022. When analyzing availability, the median period from leukapheresis to infusion for idecabtagene vicleucel was 40 days (range 33 to 79 days). Of the 140 patients included in a phase II trial, 12 (8.6%) patients could not receive the CAR T product because of progressive disease, patient withdrawal, manufacturing issues or a decision of the treating physician [61]. New methods to produce cells are currently being developed to shorten delay [99]. BiTE, on the other hand, has the distinct benefit of being readily available (off-the-shelf).

Manufacturing may be troublesome because CARs are transduced in living cells, with a failure rate of around 10% [100], while BiTEs are recombinant soluble proteins with no risk of therapy failure due to manufacturing issues. Another factor to consider is drug variability. Different T cell subset compositions can lead to product variability; CAR CD4/CD8 cell ratio normalization is frequently conducted before or after transduction to reduce variability.

The composition and phenotype of T cells affect the persistence and exhaustion of CAR T cells. Effector T cells may have an increased cytotoxic capacity, but their sole infusion does not induce lasting effects in patients. To increase their longevity, CD4 + T cells need to be coadministered [101]. In contrast to effector T cells, central memory and stem cell memory T cells show a prolonged expansion and persistence in adoptive cell therapies. The group of Riddell in Seattle showed that their combination with CD4 + T helper cells is still of utmost importance to ensure their long-term persistence [102].

Pharmacokinetics are also different, as BiTEs have a short lifetime, one of their biggest drawbacks. As a result, several infusions are required. CAR is typically a one-time administration drug, and CAR T cells can persist for more than 10 years in some patients [103]. Before administration, CAR frequently necessitates cyclophosphamide and fludarabine lymphodepletion. Furthermore, clinicians report occasional relapses soon after lymphodepletion, which is a fairly difficult scenario to handle.

When referring to the effector cell, both treatments rely on endogenous T cells to function. While T cells in BiTE should be intact during infusion, CAR T cells must be functional during leukapheresis. T cell exhaustion and anergy are of key importance, as T cell exhaustion is a reversible side effect of both therapies. T cells regenerate during treatment-free periods (2–8 weeks) in the case of BiTE. In the case of CAR, a significantly longer period may be required (months).

Cytokine release syndrome (CRS) and immune effector cell-associated neurotoxicity syndrome (ICANS) are the two most common general side effects of BiTE and CAR T cell treatments. Because of the persistence of CAR T cells, it is challenging to “stop” or “pause” CAR if they cause toxicity. To address these side effects, immunosuppressors, such as tocilizumab and/or corticosteroids, are required. New generation CARs counteract these effects by coexpressing suicide genes or having ON/OFF switch-like properties, although other methods have also been proposed [104]. BiTE, on the other hand, can be halted without causing long-term effects due to its short half-life. Another typical side effect of CAR is prolonged and severe cytopenia, which can lead to serious infections. Because BiTE does not need lymphodepletion, infections are usually acute and mild. Depending upon the targeted antigen, other specific adverse effects can occur, either as off-target or as on-target, off-tumor responses.

## BsAbs armed T cell therapy (BAT)

A third novel T cell-based immunotherapy is BAT. With this technique, leukocytes ex vivo are coated with BsAbs after being collected through leukapheresis. As a result, cytotoxic T cells can be specifically activated and targeted against tumor-associated antigens. In RRMM, SLAMF7-targeted BATs (NCT04864522) are being studied to determine clinical their safety and efficacy. In preclinical models, BATs combined the advantages of both CAR and BiTE, showing positive anti-tumor effects, while adverse effects, such as CRS, are avoided. The use of BATs allows for more precise potency control since several factors can be controlled, including (1) the amount of BiAb used to arm the ATC, (2) the cell dose per infusion and (3) the number of infusions [105].

### Patient selection for CAR versus BiTE

Given their different toxicity profiles, expected response rates and administration modalities, patient selection for either CAR or BITE may differ. In general, patients for CAR are younger (some centers propose an age limit of 75 years) because of the required conditioning regimen and possible serious complications of infections, CRS and neurotoxicity that might necessitate hemodynamic support. Cytokine release syndrome and neurotoxicity can also be seen after BITE treatment, but it tends to be milder and can often be controlled with corticosteroids.

The anti-tumor effects obtained with CAR are impressive and have never been seen before in the context of relapsed/refractory MM. These rapid and deep responses are beneficial for patients presenting with an aggressive disease (resulting from underlying genetic aberrations) or an aggressive relapse. Moreover, they allow a treatment-free interval once treatment and an early observation for toxicities are realized. In contrast, BITE therapy requires recurrent administrations given on a weekly or biweekly basis. Probably, the intervals between two administrations can be prolonged after obtaining an excellent anti-tumor effect. But currently, no data support a delay in administration. Although the response rates seen with BiTE are not as great as with CAR, they have the advantage of being immediately available, easy to administer, and can be used in frail patients.

For lymphoma patients, a high disease burden prior to CAR is an important risk factor that may be correlated with a worse prognosis—both in terms of toxicity and clinical response [106]. Patients with a substantial disease burden, a rapidly progressive disease, and/or a bulky disease, are at risk of severe CRS. In both the KarMMa and CARTITUDE-1 studies, a bridging therapy was given to the majority of patients. These bridging therapies should be personalized to each patient, according to previous lines of treatment, disease characteristics and preexisting toxicities. Of note, the presence of extramedullary disease, high-risk cytogenetics or advanced disease (ISS = 3) was no longer associated with decreased response rates in the CARTITUDE-1 study; but disease progression was observed earlier (13 months for EMD or ISS3, 20 months for high-risk cytogenetics), and not reached for the overall study population [107].

Results of the CARTITUDE-1 trials showed that for a single cilta-cel infusion of 0.75 × 106 CARs pe kg, the anti-tumor effect is significant. Thus, the OS was 97%, with 67% achieving CR. The anti-tumor effect was fast as the median time to first response was one month following infusion, while the median time to best response was 2.6 months. The median PFS was not reached, and the overall 12-month PFS was 77%. 41% of patients were not evaluable for MRD due to the lack of an identifiable clone in the baseline bone marrow sample, suggesting a deep anti-tumor response.

## Conclusion and take-home messages

This manuscript focuses on the clinical features of CAR and BiTE in MM. We depict MM antigens now being clinically studied in MM. However, it is worth noting that clinical studies on a variety of additional possible antigens may be conducted in the future (cancer testis antigens, CD70, CD126, CD229). In fact, in the future, CARs and BiTEs might be optimized to improve toxicity management, lengthen half-life or persistence and boost specificity and effectiveness. New clinical studies should be conducted to see if combining BiTE with CAR can improve ORR. If this is the case, the optimal timepoint for BiTEs administration following CAR should be evaluated. Using NK cells instead of T helper cells might improve the efficacy of CARs [108]. These T cell-engaging therapies have unseen response rates in relapsed and end-stage MM and may induce prolonged progression-free survival. Future studies that focus on their use in earlier treatment lines or in different patient populations will try to define their optimal use, especially whether these constructs could be optimized to a point that a transplant might no longer be required in the future.

Table 4 summarizes the advantages versus the disadvantages of both strategies.

**Table 4:** CAR versus BiTE in MM

	Car	BiTE	Bat
Advantages	Strong and rapid anti-tumor effects	Off-the-shelf available	Strong and rapid anti-tumor effects
	Efficient in different subgroups	Good anti-tumor control	Potency control
	Autologous or allogeneic products	Dosing can be stopped in case of adverse effects	
Disadvantages	Delay in production	Continuous treatment	Delay in production
	Side effects	Costs + +	costs (?)
	costs + + +		

## Data Availability

Not applicable.

## Abbreviations

CAR: Chimeric antigen receptor T cell therapy
CARs: Chimeric antigen receptors
BITE: Bispecific T cell engager therapy
BiTEs: Bispecific T cell engagers
MM: Multiple myeloma
MGUS: Monoclonal gammopathy of undetermined significance
PI: Proteasome inhibitors
IMiDs: Immunomodulatory drugs
HDACi: Histone deacetylase inhibitors
mABs: Monoclonal antibodies
ADC: Antibody–drug conjugates
SINEs: Selective inhibitors of nuclear export
SCT: Stem cell transplantation
R/R: Relapsed/refractory
RRMM: Relapsed/refractory multiple myeloma
BsAbs: Bispecific antibodies
IgG: Immunoglobulin G
*V*_*H*_: Variable heavy chain
*V*_*L*_: Variable light chain
*F*_ab_: Antigen-binding fragments
*C*_*H*_: Constant heavy chain
*C*_*L*_: Constant light chain
VHH: Heavy chains with a single variable region
scFv: Single-chain variable fragments
TCR: T cell receptor
BCMA: B cell maturation antigen
ORR: Overall response rate
PFS: Progression-free survival
OS: Overall survival
TACI: Transmembrane activator and calcium modulator and cyclophilin ligand interactor
BAFF: B cell activating factor
APRIL: A proliferation-inducing ligand
SLAMF7: Signaling lymphocyte activation molecule family receptor 7
NKG2D: Natural killer group 2, member D
FcRH5: Fc receptor-like 5

## References

[1] Padala SA, Barsouk A, Barsouk A, Rawla P, Vakiti A, Kolhe R (2021). Epidemiology, staging, and management of multiple myeloma. Med Sci.

[2] Kumar SK, Rajkumar V, Kyle RA, van Duin M, Sonneveld P, Mateos MV (2017). Multiple myeloma. Nat Rev Dis Primers.

[3] Casey M, Nakamura K (2021). The cancer-immunity cycle in multiple myeloma. Immuno Targets Ther.

[4] Dimopoulos MA, Moreau P, Terpos E, Mateos M, Zweegman S, Cook G (2021). Multiple myeloma: EHA-ESMO clinical practice guidelines for diagnosis, treatment and follow-up†. Ann Oncol.

[5] Ramasamy K, Gay F, Weisel K, Zweegman S, Mateos MV, Richardson P (2021). Improving outcomes for patients with relapsed multiple myeloma: challenges and considerations of current and emerging treatment options. Blood Rev.

[6] Cavo M, Gay F, Beksac M, Pantani L, Petrucci MT, Dimopoulos MA (2020). Autologous haematopoietic stem-cell transplantation versus bortezomib–melphalan–prednisone, with or without bortezomib–lenalidomide–dexamethasone consolidation therapy, and lenalidomide maintenance for newly diagnosed multiple myeloma (EMN02/HO95): a mult. Lancet Haematol.

[7] Davis LN, Sherbenou DW (2021). Emerging therapeutic strategies to overcome drug resistance in multiple myeloma. Cancers (Basel).

[8] Uckun FM (2021). Overcoming the immunosuppressive tumor microenvironment in multiple myeloma. Cancers (Basel).

[9] Du J, Zhuang J (2021). Major advances in the treatment of multiple myeloma in American Society of Hematology annual meeting 2020. Chronic Dis Transl Med.

[10] Gross G, Waks T, Eshhar Z (1989). Expression of immunoglobulin-T-cell receptor chimeric molecules as functional receptors with antibody-type specificity. Proc Natl Acad Sci.

[11] Mo F, Mamonkin M (2020). Generation of chimeric antigen receptor t cells using gammaretroviral vectors. Methods Mol Biol.

[12] Eyquem J, Mansilla-Soto J, Giavridis T, van der Stegen SJC, Hamieh M, Cunanan KM (2017). Targeting a CAR to the TRAC locus with CRISPR/Cas9 enhances tumour rejection. Nature.

[13] Bishop DC, Caproni L, Gowrishankar K, Legiewicz M, Karbowniczek K, Tite J (2020). CAR T cell generation by piggybac transposition from linear doggybone DNA vectors requires transposon DNA-flanking regions. Mol Ther Methods Clin Dev.

[14] Chicaybam L, Abdo L, Bonamino MH (2020). Generation of CAR+ T lymphocytes using the sleeping beauty transposon system. Methods Mol Biol.

[15] Parayath NN, Stephan SB, Koehne AL, Nelson PS, Stephan MT (2020). In vitro-transcribed antigen receptor mRNA nanocarriers for transient expression in circulating T cells in vivo. Nat Commun.

[16] Lin L, Cho SF, Xing L, Wen K, Li Y, Yu T (2021). Preclinical evaluation of CD8+ anti-BCMA mRNA CAR T cells for treatment of multiple myeloma. Leukemia.

[17] Foster JB, Choudhari N, Perazzelli J, Storm J, Hofmann TJ, Jain P (2019). Purification of mRNA encoding chimeric antigen receptor is critical for generation of a robust T-Cell response. Hum Gene Ther.

[18] Yang EY, Shah K (2020). Nanobodies: next generation of cancer diagnostics and therapeutics. Front Oncol.

[19] Branella GM, Spencer HT. Targeted Cell Killing; 2022.

[20] Fujiwara K, Tsunei A, Kusabuka H, Ogaki E, Tachibana M, Okada N (2020). Hinge and transmembrane domains of chimeric antigen receptor regulate receptor expression and signaling threshold. Cells.

[21] Stornaiuolo A, Valentinis B, Sirini C, Scavullo C, Asperti C, Zhou D (2021). Characterization and functional analysis of CD44v6.CAR T cells endowed with a new low-affinity nerve growth factor receptor-based spacer. Hum Gene Ther.

[22] Jayaraman J, Mellody MP, Hou AJ, Desai RP, Fung AW, Pham AHT (2020). CAR-T design: elements and their synergistic function. EBioMedicine..

[23] Tomuleasa C, Fuji S, Berce C, Onaciu A, Chira S, Petrushev B (2018). Chimeric antigen receptor T-cells for the treatment of B-cell acute lymphoblastic leukemia. Front Immunol.

[24] Tat T, Li H, Constantinescu CS, Onaciu A, Chira S, Osan C (2018). Genetically enhanced T lymphocytes and the intensive care unit. Oncotarget.

[25] Sterner RC, Sterner RM (2021). CAR-T cell therapy: current limitations and potential strategies. Blood Cancer J.

[26] Abate-Daga D, Davila ML (2016). CAR models: next-generation CAR modifications for enhanced T-cell function. Mol Ther Oncol.

[27] Weinkove R, George P, Dasyam N, McLellan AD (2019). Selecting costimulatory domains for chimeric antigen receptors: functional and clinical considerations. Clin Transl Immunol.

[28] Huang R, Li X, He Y, Zhu W, Gao L, Liu Y (2020). Recent advances in CAR-T cell engineering. J Hematol Oncol.

[29] Duan D, Wang K, Wei C, Feng D, Liu Y, He Q (2021). The BCMA-Targeted Fourth-Generation CAR-T Cells Secreting IL-7 and CCL19 for therapy of refractory/recurrent multiple myeloma. Front Immunol.

[30] Cells CART, Benmebarek M reda, Karches CH, Cadilha BL, Lesch S, Endres S, et al. Killing Mechanisms of Chimeric Antigen Receptor; 2019. 10.3390/ijms20061283 PMC6470706 30875739

[31] Lejeune M, Köse MC, Duray E, Einsele H, Beguin Y, Caers J (2020). Bispecific, T-cell-recruiting antibodies in B-cell malignancies. Front Immunol.

[32] Mack M, Riethmuller G, Kufer P (1995). A small bispecific antibody construct expressed as a functional single- chain molecule with high tumor cell cytotoxicity. Proc Natl Acad Sci U S A.

[33] Tian Z, Liu M, Zhang Y, Wang X (2021). Bispecific T cell engagers: an emerging therapy for management of hematologic malignancies. J Hematol Oncol.

[34] Santich BH, Park JA, Tran H, Guo HF, Huse M, Cheung NKV (2020). Interdomain spacing and spatial configuration drive the potency of IgG-[L]-scFv T cell bispecific antibodies. Sci Transl Med.

[35] Bu DX, Singh R, Choi EE, Ruella M, Cruz SN, Mansfield KG (2018). Pre-clinical validation of B cell maturation antigen (BCMA) as a target for T cell immunotherapy of multiple myeloma. Oncotarget.

[36] Carpenter RO, Evbuomwan MO, Pittaluga S, Rose JJ, Raffeld M, Yang S (2013). B-cell maturation antigen is a promising target for adoptive T-cell therapy of multiple myeloma. Clin Cancer Res.

[37] Brudno JN, Maric I, Hartman SD, Rose JJ, Wang M, Lam N (2018). T cells genetically modified to express an anti–B-Cell maturation antigen chimeric antigen receptor cause remissions of poor-prognosis relapsed multiple myeloma. J Clin Oncol.

[38] Zhang L, Shen X, Yu W, Li J, Zhang J, Zhang R (2021). Comprehensive meta-analysis of anti-BCMA chimeric antigen receptor T-cell therapy in relapsed or refractory multiple myeloma. Ann Med.

[39] Lin L, Cho SF, Xing L, Wen K, Li Y, Yu T (2021). Preclinical evaluation of CD8+ anti-BCMA mRNA CAR T cells for treatment of multiple myeloma. Leukemia.

[40] Costello C, Derman BA, Kocoglu MH, Deol A, Ali AA, Gregory T (2021). Clinical trials of BCMA-targeted CAR-T cells utilizing a novel non-viral transposon system. Blood.

[41] Costello CL, Cohen AD, Patel KK, Ali SS, Berdeja JG, Shah N (2020). Phase 1/2 study of the safety and response of P-BCMA-101 CAR-T cells in patients with relapsed/refractory (r/r) multiple myeloma (mm) (prime) with novel therapeutic strategies. Blood.

[42] Costello CL, Gregory TK, Ali SA, Berdeja JG, Patel KK, Shah ND (2019). Phase 2 study of the response and safety of P-Bcma-101 CAR-T cells in patients with relapsed/refractory (r/r) multiple myeloma (MM) (PRIME). Blood.

[43] Ivics Z (2021). Potent CAR-T cells engineered with sleeping beauty transposon vectors display a central memory phenotype. Gene Ther.

[44] Green DJ, Pont M, Sather BD, Cowan AJ, Turtle CJ, Till BG (2018). Fully human Bcma targeted chimeric antigen receptor t cells administered in a defined composition demonstrate potency at low doses in advanced stage high risk multiple myeloma. Blood.

[45] Rodríguez-Lobato LG, Ganzetti M, Fernández de Larrea C, Hudecek M, Einsele H, Danhof S (2020). CAR T-cells in multiple myeloma: state of the art and future directions. Front Oncol.

[46] Cohen AD, Garfall AL, Stadtmauer EA, Melenhorst JJ, Lacey SF, Lancaster E (2019). B cell maturation antigen–specific CAR T cells are clinically active in multiple myeloma. J Clin Investig.

[47] Green DJ, Pont M, Cowan AJ, Cole GO, Sather BD, Nagengast AM (2019). Response to Bcma CAR-T cells correlates with pretreatment target antigen density and is improved by small molecule inhibition of gamma secretase. Blood.

[48] van de Donk NWCJ, Themeli M, Usmani SZ (2021). Determinants of response and mechanisms of resistance of CAR T-cell therapy in multiple myeloma. Cancer Discov.

[49] Seipel K, Porret N, Wiedemann G, Jeker B, Bacher VU, Pabst T (2022). sBCMA plasma level dynamics and anti-BCMA CAR-T-Cell treatment in relapsed multiple myeloma. Curr Issues Mol Biol.

[50] Jiang Hua, Dong Baoxia, Gao Li, Liu Li, Ge Jian, He Aili (2021). Long-term follow-up results of a multicenter first-in-human study of the dual BCMA/CD19 Targeted FasT CAR-T GC012F for patients with relapsed/refractory multiple myeloma. J Clin.

[51] Zhang H, Gao L, Liu L, Wang J, Wang S, Gao L (2019). A Bcma and CD19 bispecific CAR-T for relapsed and refractory multiple myeloma. Blood.

[52] Wudhikarn K, Mailankody S, Smith EL (2020). Future of CAR T cells in multiple myeloma. Hematology.

[53] van der Schans JJ, van de Donk NWCJ, Mutis T (2020). Dual targeting to overcome current challenges in multiple myeloma car T-cell treatment. Front Oncol.

[54] Laurent SA, Hoffmann FS, Kuhn PH, Cheng Q, Chu Y, Schmidt-Supprian M (2015). γ-secretase directly sheds the survival receptor BCMA from plasma cells. Nat Commun.

[55] Pont MJ, Hill T, Cole GO, Abbott JJ, Kelliher J, Salter AI (2019). γ-Secretase inhibition increases efficacy of BCMA-specific chimeric antigen receptor T cells in multiple myeloma. Blood.

[56] Lin Q, Zhao J, Song Y, Liu D (2019). Recent updates on CAR T clinical trials for multiple myeloma. Mol Cancer.

[57] Sommer C, Boldajipour B, Kuo TC, Bentley T, Sutton J, Chen A (2019). Preclinical evaluation of allogeneic CAR T cells targeting BCMA for the treatment of multiple myeloma. Mol Ther.

[58] Mikkilineni L, Manasanch EE, Lam N, Vanasse D, Brudno JN, Maric I (2019). T Cells expressing an anti-B-cell maturation antigen (BCMA) chimeric antigen receptor with a fully-human heavy-chain-only antigen recognition domain induce remissions in patients with relapsed multiple myeloma. Blood.

[59] Bianchi ME, Mezzapelle R (2020). The chemokine receptor CXCR4 in cell proliferation and tissue regeneration. Front Immunol.

[60] Wu X, Shi B, Zhang J, Shi Z, Di S, Fan M (2017). A fusion receptor as a safety switch, detection, and purification biomarker for adoptive transferred T cells. Mol Ther.

[61] Munshi NC, Anderson LDJ, Shah N, Madduri D, Berdeja J, Lonial S (2021). Idecabtagene vicleucel in relapsed and refractory multiple myeloma. N Engl J Med.

[62] Stauffer A, Ray C, Hall M (2021). A flexible multiplatform bioanalytical strategy for measurement of total circulating shed target receptors: application to soluble b cell maturation antigen levels in the presence of a bispecific antibody drug. Assay Drug Dev Technol.

[63] Tai YT, Anderson KC (2015). Targeting B-cell maturation antigen in multiple myeloma. Immunotherapy.

[64] Lee L, Draper B, Chaplin N, Philip B, Chin M, Galas-Filipowicz D (2018). An APRIL-based chimeric antigen receptor for dual targeting of BCMA and TACI in multiple myeloma. Blood.

[65] Schmidts A, Ormhøj M, Choi BD, Taylor AO, Bouffard AA, Scarfò I (2019). Rational design of a trimeric April-based CAR-binding domain enables efficient targeting of multiple myeloma. Blood Adv.

[66] Drent E, Groen RWJ, Noort WA, Themeli M, van Bueren JJL, Parren PWHI (2016). Pre-clinical evaluation of CD38 chimeric antigen receptor engineered T cells for the treatment of multiple myeloma. Haematologica.

[67] Zuch de Zafra CL, Fajardo F, Zhong W, Bernett MJ, Muchhal US, Moore GL (2019). Targeting multiple myeloma with AMG 424, a novel anti-CD38/CD3 bispecific T-cell-recruiting antibody optimized for cytotoxicity and cytokine release. Clin Cancer Res Off J Am Assoc Cancer Res.

[68] Richter JR, Landgren CO, Kauh JS, Back J, Salhi Y, Reddy V (2018). Phase 1, multicenter, open-label study of single-agent bispecific antibody t-cell engager GBR 1342 in relapsed/refractory multiple myeloma. J Clin Oncol.

[69] Figure OS (2021). Bi38–3 is a novel CD38 / CD3 bispecific T-cell engager with low toxicity for the treatment of multiple myeloma. Haematologica.

[70] Hsi ED, Steinle R, Balasa B, Szmania S, Draksharapu A, Shum BP, Huseni M, Powers D, Nanisetti A, Zhang Y, Rice AG, van Abbema A, Wong M, Liu G, Zhan F, Dillon M, Chen S (2016). Su and MBW CS1, a potential new therapeutic antibody target for the treatment of multiple myeloma. Physiol Behav.

[71] Kikuchi J, Hori M, Iha H, Toyama-Sorimachi N, Hagiwara S, Kuroda Y (2020). Soluble SLAMF7 promotes the growth of myeloma cells via homophilic interaction with surface SLAMF7. Leukemia.

[72] Albinger N, Hartmann J, Ullrich E (2021). Current status and perspective of CAR-T and CAR-NK cell therapy trials in Germany. Gene Ther..

[73] Smith EL, Harrington K, Staehr M, Masakayan R, Jones J, Long TJ, et al. GPRC5D is a target for the immunotherapy of multiple myeloma with rationally designed CAR T cells. Sci Transl Med. 2019;11(485). 10.1126/scitranslmed.aau7746 PMC7508042 30918115

[74] Atamaniuk J, Gleiss A, Porpaczy E, Kainz B, Grunt TW, Raderer M (2012). Overexpression of G protein-coupled receptor 5D in the bone marrow is associated with poor prognosis in patients with multiple myeloma. Eur J Clin Invest.

[75] Berdeja JG, Krishnan AY, Oriol A, van de Donk NWCJ, Rodríguez-Otero P, Askari E (2021). Updated results of a phase 1, first-in-human study of talquetamab, a G protein-coupled receptor family C group 5 member D (GPRC5D) × CD3 bispecific antibody, in relapsed/refractory multiple myeloma (MM). J Clin Oncol.

[76] Elkins K, Zheng B, Go MA, Slaga D, Du C, Scales SJ (2012). FcRL5 as a target of antibody-drug conjugates for the treatment of multiple myeloma. Mol Cancer Ther.

[77] O’Connell FP, Pinkus JL, Pinkus GS (2004). CD138 (Syndecan-1), a plasma cell marker: immunohistochemical profile in hematopoietic and nonhematopoietic neoplasms. Am J Clin Pathol.

[78] Guo B, Chen M, Han Q, Hui F, Dai H, Zhang W (2016). CD138-directed adoptive immunotherapy of chimeric antigen receptor (CAR)-modified T cells for multiple myeloma. J Cell Immunother.

[79] Ishikawa H, Tsuyama N, Mahmoud MS, Fujii R, Abroun S, Liu S (2002). CD19 expression and growth inhibition of tumours in human multiple myeloma. Leuk Lymphoma.

[80] Kellner J, Wallace C, Liu B, Li Z (2019). Definition of a multiple myeloma progenitor population in mice driven by enforced expression of XBP1s. JCI Insight.

[81] Garfall AL, Stadtmauer EA, Hwang WT, Lacey SF, Melenhorst JJ, Krevvata M (2018). Anti-CD19 CAR t cells with high-dose melphalan and autologous stem cell transplantation for refractory multiple myeloma. JCI Insight.

[82] Rodríguez-Otero P, Prósper F, Alfonso A, Paiva B, San Miguel JF (2020). Car t-cells in multiple myeloma are ready for prime time. J Clin Med.

[83] Sidana S, Shah N (2019). CAR T-cell therapy: Is it prime time in myeloma?. Hematol (United States).

[84] Wensveen FM, Jelenčić V, Polić B (2018). NKG2D: a master regulator of immune cell responsiveness. Front Immunol.

[85] Nikiforow S, Werner L, Murad J, Jacobs M, Johnston L, Patches S (2016). Safety data from a first-in-human phase 1 Trial of NKG2D chimeric antigen receptor-T Cells in AML/MDS and multiple myeloma. Blood.

[86] Sallman DA, Brayer JB, Poire X, Havelange V, Awada A, Lewalle P (2019). Results from the completed dose-escalation of the hematological arm of the phase i think study evaluating multiple infusions of NKG2D-based CAR T-cells as standalone therapy in relapse/refractory acute myeloid leukemia and myelodysplastic syndrome patient. Blood.

[87] van Camp BB, Durie BGM, Spier C, de Waele M, van Riet I, Vela E (1990). Plasma cells in multiple myeloma express a natural killer cell-associated antigen CD56 (NKH-1; Leu-19). BLOOD.

[88] Neri P, Ren L, Azab AK, Brentnall M, Gratton K, Klimowicz AC (2011). Integrin β7-mediated regulation of multiple myeloma cell adhesion, migration, and invasion. Blood.

[89] Hosen N, Matsunaga Y, Hasegawa K, Matsuno H, Nakamura Y, Makita M (2017). The activated conformation of integrin β7 is a novel multiple myeloma-specific target for CAR T cell therapy. Nat Med.

[90] Liebisch P, Eppinger S, Schöpflin C, Stehle G, Munzert G, Döhner H (2005). CD44v6, a target for novel antibody treatment approaches, is frequently expressed in multiple myeloma and associated with deletion of chromosome arm 13q. Haematologica.

[91] Ghafouri-Fard S, Seifi-Alan M, Shamsi R, Esfandiary A (2015). Immunotherapy in multiple myeloma using cancer-testis antigens. Int J Cancer Manag.

[92] Ghafouri-Fard S (2015). Expression of cancer-testis antigens in stem cells: is it a potential drawback or an advantage in cancer immunotherapy. Asian Pac J Cancer Prev.

[93] Zhang W, Qu X, Chen B, Snyder M, Li B, Tang Y (2016). Engineered CAR T Cells targeting the cancer-associated tn-glycoform of the membrane mucin MUC1 control. Immunity.

[94] Gutierrez R, Shah PD, Hamid O, Garfall AL, Posey A, Bishop MR (2021). Phase I experience with first in class TnMUC1 targeted chimeric antigen receptor T-cells in patients with advanced TnMUC1 positive solid tumors. J Clin Oncol.

[95] Tamura H, Ishibashi M, Sunakawa-Kii M, Inokuchi K (2020). PD-L1-PD-1 pathway in the pathophysiology of multiple myeloma. Cancers (Basel).

[96] Borgert R,BCOP P. Improving outcomes and mitigating costs associated with CAR T-Cell therapy. Supplements and Featured Publications. 2021;27(13). Available from: https://cdn.sanity.io/files/0vv8moc6/ajmc/cf06f4909c69be1f7818ab8337ccd598619c1966.pdf 10.37765/ajmc.2021.88737 34407361

[97] Subklewe M (2021). BiTEs better than CAR T cells. Blood Adv.

[98] Thielen FW, van Dongen-Leunis A, Arons AMM, Ladestein JR, Hoogerbrugge PM, Uyl-deGroot CA (2020). Cost-effectiveness of Anti-CD19 chimeric antigen receptor T-Cell therapy in pediatric relapsed/refractory B-cell acute lymphoblastic leukemia. A societal view. Eur J Haematol.

[99] Maschan M, Caimi PF, Reese-Koc J, Sanchez GP, Sharma AA, Molostova O (2021). Multiple site place-of-care manufactured anti-CD19 CAR-T cells induce high remission rates in B-cell malignancy patients. Nat Commun.

[100] Majzner RG, Mackall CL (2019). Clinical lessons learned from the first leg of the CAR T cell journey. Nat Med.

[101] Marzo AL, Kinnear BF, Lake RA, Frelinger JJ, Collins EJ, Robinson BW (2000). Tumor-specific CD4+ T cells have a major “post-licensing” role in CTL mediated anti-tumor immunity. J Immunol.

[102] Melenhorst JJ, Chen GM, Wang M, Porter DL, Chen C, Collins MA (2022). Decade-long leukaemia remissions with persistence of CD4(+) CAR T cells. Nature.

[103] Brandt LJB, Barnkob MB, Michaels YS, Heiselberg J, Barington T (2020). Emerging approaches for regulation and control of CAR T Cells: a mini review. Front Immunol.

[104] Lum LG, Thakur A, Elhakiem A, Alameer L, Dinning E, Huang M (2020). Anti-CS1 × Anti-CD3 bispecific antibody (BiAb)-armed anti-CD3 activated T cells (CS1-BATs) kill CS1+ myeloma cells and release type-1 cytokines. Front Oncol.

[105] Amini L, Silbert SK, Maude SL, Nastoupil LJ, Ramos CA, Brentjens RJ (2022). Preparing for CAR T cell therapy: patient selection, bridging therapies and lymphodepletion. Nat Rev Clin Oncol.

[106] Martin T, Usmani SZ, Berdeja JG, Jakubowiak A, Agha M, Cohen AD (2021). Updated results from CARTITUDE-1: Phase 1b/2Study of ciltacabtagene autoleucel, a B-cell maturation antigen-directed chimeric antigen receptor T cell therapy, in patients with relapsed/refractory multiple myeloma. Blood.

[107] Xie G, Dong H, Liang Y, Ham JD, Rizwan R, Chen J. CAR-NK cells: A promising cellular immunotherapy for cancer. EBioMedicine. 2020;59. 10.1016/j.ebiom.2020.102975 PMC7452675 32853984

[108] Qian Y, Qian Z, Zhao X, Pan W, Wei X, Meng H (2021). Successful treatment of relapsed/refractory extramedullary multiple myeloma with anti-bcma CAR-t cell therapy followed by haploidentical hematopoietic stem cell transplantation: a case report and a review of the contemporary literature. Front Med..

[109] Lin Y, Raje NS, Berdeja JG, Siegel DS, Jagannath S, Madduri D (2020). Idecabtagene vicleucel (ide-cel, bb2121), a BCMA-directed CAR T cell therapy, in patients with relapsed and refractory multiple myeloma: updated results from phase 1 CRB-401 study. Blood.

[110] Usmani SZ, Berdeja JG, Truppel-Hartmann A, Fei Y, Wortman-Vayn H, Shelat S (2021). KarMMa-4: Idecabtagene vicleucel (ide-cel, bb2121), a BCMA-directed CAR T-cell therapy in high-risk newly diagnosed multiple myeloma. J Clin Oncol.

[111] Raje NS, Shah N, Jagannath S, Kaufman JL, Siegel DS, Munshi NC (2021). Updated clinical and correlative results from the phase I CRB-402 study of the BCMA-targeted CAR T cell therapy bb21217 in patients with relapsed and refractory multiple myeloma. Blood.

[112] Kumar SK, Baz RC, Orlowski RZ, Anderson LD, Ma H, Shrewsbury A (2020). Results from lummicar-2: a phase 1b/2 study of fully human B-cell maturation antigen-specific CAR T Cells (CT053) in patients with relapsed and/or refractory multiple myeloma. Blood.

[113] Jie J, Hao S, Jiang S, Li Z, Yang M, Zhang W (2019). Phase 1 trial of the safety and efficacy of fully human anti-Bcma CAR T cells in relapsed/refractory multiple myeloma. Blood.

[114] An G, Sui W, Wang T, Qu X, Zhang X, Yang J (2020). An anti-Bcma CAR T-cell therapy (C-CAR088) shows promising safety and efficacy profile in relapsed or refractory multiple myeloma. Blood.

[115] Han L, Gao Q, Zhou K, Zhou J, Yin QS, Fang B (2021). The clinical study of anti-BCMA CAR-T with single-domain antibody as antigen binding domain. J Clin Oncol.

[116] Liu Y, Chen Z, Wei R, Shi L, He F, Shi Z (2018). Remission observed from a phase 1 clinical study of CAR-T therapy with safety switch targeting BCMA for patients with relapsed/refractory multiple myeloma. J Clin Oncol.

[117] Sperling AS, Nikiforow S, Nadeem O, Mo CC, Laubach JP, Anderson KC (2021). Phase I study of PHE885, a fully human BCMA-directed CAR-T cell therapy for relapsed/refractory multiple myeloma manufactured in <2 days using the T-charge TM platform. Blood.

[118] Mailankody S, Ghosh A, Staehr M, Purdon TJ, Roshal M, Halton E (2018). Clinical responses and pharmacokinetics of MCARH171, a human-derived Bcma targeted CAR T cell therapy in relapsed/refractory multiple myeloma: final results of a phase I clinical trial. Blood.

[119] Jiang H, Dong B, Gao L, Liu L, Ge J, He A (2021). Long-term follow-up results of a multicenter first-in-human study of the dual BCMA/CD19 Targeted FasT CAR-T GC012F for patients with relapsed/refractory multiple myeloma. J Clin Oncol.

[120] Popat R, Zweegman S, Cavet J, Yong K, Lee L, Faulkner J (2019). Phase 1 first-in-human study of AUTO2, the first chimeric antigen receptor (CAR) T cell targeting APRIL for Patients with relapsed/refractory multiple myeloma (RRMM). Blood.

[121] Usmani SZ, Berdeja JG, Madduri D, Jakubowiak AJ, Agha ME, Cohen AD (2021). Ciltacabtagene autoleucel, a B-cell maturation antigen (BCMA)-directed chimeric antigen receptor T-cell (CAR-T) therapy, in relapsed/refractory multiple myeloma (R/R MM): Updated results from CARTITUDE-1. J Clin Oncol.

[122] Zhao WH, Liu J, Wang BY, Chen YX, Cao XM, Yang Y (2018). A phase 1, open-label study of LCAR-B38M, a chimeric antigen receptor T cell therapy directed against B cell maturation antigen, in patients with relapsed or refractory multiple myeloma. J Hematol Oncol.

[123] Mailankody S, Jakubowiak AJ, Htut M, Costa LJ, Lee K, Ganguly S (2020). Orvacabtagene autoleucel (orva-cel), a B-cell maturation antigen (BCMA)-directed CAR T cell therapy for patients (pts) with relapsed/refractory multiple myeloma (RRMM): update of the phase 1/2 EVOLVE study (NCT03430011). J Clin Oncol.

[124] Kumar SK, Baz RC, Orlowski RZ, Anderson LD, Ma H, Shrewsbury A (2020). Results from lummicar-2: a phase 1b/2 study of fully human B-cell maturation antigen-specific CAR T cells (CT053) in patients with relapsed and/or refractory multiple myeloma. Blood.

[125] Oliver-Caldes A, Jiménez R, Español-Rego M, Cibeira MT, Ortiz-Maldonado V, Quintana LF (2021). First report of CART treatment in AL amyloidosis and relapsed/refractory multiple myeloma. J Immunother Cancer.

[126] Ma F, Li J, Ren J, Zhang X, Wang F, Guo X (2018). Preliminary results of phase I/II study of SENL-B19 chimeric antigen receptor T cell therapy in pediatric and adult patients with relapsed/refractory acute lymphoblastic leukemia (r/r-ALL). Ann Oncol.

[127] Zhang Y, Zhang C, Zhou J, Zhang J, Chen X, Chen J (2021). Case report: reversible neurotoxicity and a clinical response induced by BCMA-directed chimeric antigen receptor T Cells against multiple myeloma with central nervous system involvement. Front Immunol.

[128] Jagannath S, Lin Y, Goldschmidt H, Reece D, Nooka A, Senin A (2021). KarMMa-RW: comparison of idecabtagene vicleucel with real-world outcomes in relapsed and refractory multiple myeloma. Blood Cancer J.

[129] Agha ME, Cohen AD, Madduri D, Cohen YC, Delforge M, Hillengass J (2021). CARTITUDE-2: Efficacy and safety of ciltacabtagene autoleucel (cilta-cel), a BCMA-directed CAR T-cell therapy, in patients with progressive multiple myeloma (MM) after one to three prior lines of therapy. J Clin Oncol.

[130] Delforge M, Baz RC, Cavo M, Callander NS, Ghobadi A, Rodriguez-Otero P (2020). KarMMa-3: a phase 3 study of idecabtagene vicleucel (ide-cel, bb2121), a BCMA-directed CAR T cell therapy vs standard regimens in relapsed and refractory multiple myeloma. Blood.

[131] Topp MS, Duell J, Zugmaier G, Attal M, Moreau P, Langer C (2020). Anti–B-cell maturation antigen bite molecule AMG 420 induces responses in multiple myeloma. J Clin Oncol.

[132] Bahlis NJ, Raje NS, Costello C, Dholaria BR, Solh MM, Levy MY (2021). Efficacy and safety of elranatamab (PF-06863135), a B-cell maturation antigen (BCMA)-CD3 bispecific antibody, in patients with relapsed or refractory multiple myeloma (MM). J Clin Oncol.

[133] Garfall AL, Usmani SZ, Mateos MV, Nahi H, van de Donk NWCJ, San-Miguel JF (2020). Updated phase 1 results of teclistamab, a B-Cell maturation antigen (BCMA) x CD3 bispecific antibody, in relapsed and/or refractory multiple myeloma (RRMM). Blood.

[134] Rodriguez-Otero P, Dholaria B, Askari E, Reece DE, van de Donk NWCJ, Chari A (2021). Subcutaneous teclistamab in combination with daratumumab for the treatment of patients with relapsed/refractory multiple myeloma: results from a phase 1b multicohort study. Blood.

[135] Costa LJ, Wong SW, Bermúdez A, de la Rubia J, Mateos MV, Ocio EM (2019). First clinical study of the B-cell maturation antigen (BCMA) 2+1 T cell engager (TCE) CC-93269 in patients (Pts) with relapsed/refractory multiple myeloma (RRMM): interim results of a phase 1 multicenter trial. Blood.

[136] Rodriguez C, D’Souza A, Shah N, Voorhees PM, Buelow B, Vij R (2020). Initial Results of a phase I study of TNB-383B, a BCMA x CD3 bispecific T-cell redirecting antibody relapsed/refractory multiple myeloma. Blood.

[137] Harrison SJ, Minnema MC, Lee HC, Spencer A, Kapoor P, Madduri D (2020). A Phase 1 first in human (FIH) study of AMG 701, an anti-B-cell maturation antigen (BCMA) half-life extended (HLE) BiTE® (bispecific T-cell engager) molecule, in relapsed/refractory (RR) multiple myeloma (MM). Blood.

[138] Madduri D, Rosko A, Brayer J, Zonder J, Bensinger WI, Li J (2020). REGN5458, a BCMA x CD3 bispecific monoclonal antibody, induces deep and durable responses in patients with relapsed/refractory multiple myeloma (RRMM). Blood.

[139] Moreau P, Usmani SZ, Garfall AL, van de Donk NWCJ, Nahi H, San-Miguel J (2021). Updated results from MajesTec-1: phase 1/2 study of teclistamab, a B-cell maturation antigen x cd3 bispecific antibody relapsed/refractory multiple myeloma. Blood.

[140] Cohen AD, Harrison SJ, Krishnan A, Fonseca R, Forsberg PA, Spencer A (2020). Initial clinical activity and safety of BFCR4350A, a FcRH5/CD3 T-cell-engaging bispecific antibody relapsed/refractory multiple myeloma. Blood.

[141] Constantinescu C, Pasca S, Tat T, Teodorescu P, Vlad C, Iluta S, Dima D, Tomescu D, Scarlatescu E, Tanase A, Sigurjonsson OE, Colita A, Einsele H, Tomuleasa C (2020). Continuous renal replacement therapy in cytokine release syndrome following immunotherapy or cellular therapies?. J Immunother Cancer..

